# Interference Pattern Caused by Bilateral Bone Conduction Stimulation Impairs Sound Localization

**DOI:** 10.1002/advs.202500302

**Published:** 2025-06-10

**Authors:** Liu‐Jie Ren, Yi Yu, Cheng Hua, You‐Zhou Xie, Wen‐Juan Yao, Jun‐Yi Liang, Chen‐Long Li, Tian‐Yu Zhang

**Affiliations:** ^1^ FPRS Department / ENT Institute / NHC Key Laboratory of Hearing Medicine Eye & ENT Hospital of Fudan University Shanghai 200031 China; ^2^ College of Medical Instruments Shanghai University of Medicine & Health Science Shanghai 201318 China; ^3^ Department of Aeronautics and Astronautics Fudan University Shanghai 200433 China; ^4^ School of Mechanics and Engineering Science Shanghai University Shanghai 200444 China; ^5^ Genomic Medicine Institute, Cleveland Clinic Foundation Cleveland OH 44106 USA

**Keywords:** bone conduction, crosstalk, psychoacoustics, sound localization, wave interference

## Abstract

While bilateral fitting of bone conduction hearing devices (BCHDs) enhances spatial hearing, further improvements are constrained by the unresolved effects of crosstalk – an influential factor that disrupts binaural acoustic cues, such as interaural level difference (ILD) and interaural phase differences (IPD), essential for accurate sound localization. This paper introduces a simplified theoretical model to describe the crosstalk phenomenon and predict the cochlear vibrational responses under bilateral bone conduction (BC) based on principles of wave interference and superposition. The model reveals sound lateralization patterns across different ILD and IPD combinations, different from well‐established principles governing air conduction (AC) sound localization, including the precedence effect and intensity rule. These predicted patterns are experimentally validated through cadaveric vibration measurements and are further corroborated in psychoacoustic sound lateralization tests conducted on healthy volunteers. The findings suggest that crosstalk induces wave interference in the skull and leads to the superposition of bilateral signals at the cochleae, resulting in these atypical lateralization patterns. This evidence highlights the inherent challenges of sound localization under BC compared to AC, identifying crosstalk‐induced wave interference as a primary obstacle to improved spatial hearing for bilateral BCHD users.

## Graphical abstract

This work explores how crosstalk‐induced wave interference affects bone conduction (BC) sound localization. Using a theoretical model, cadaveric vibrometry, and psychoacoustic tests, atypical BC lateralization patterns are revealed that contradict air conduction (AC) principles. Experimental validation confirms that crosstalk disrupts binaural cues, making it a key obstacle to accurate spatial hearing with bilateral fitting of BC hearing devices.

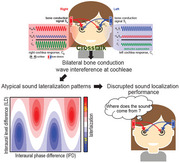

## 1 Introduction

When two coherent waves interact, they can create periodic spatial patterns, such as the classic double‐slit interference pattern, the ripples formed when two raindrops hit a pond, or standing acoustic waves in an empty room. Similarly, vibrational waves generated by bone conduction (BC) earphones or hearing devices can also interfere within the human skull.^[^ ^1^ ^]^ These waves, falling within the human auditory frequency range (100–10 000 Hz), propagate at speeds of 400–800 m s^−1^,^[^ ^2^, ^3^, ^4^ ^]^ before reaching the cochleae – the natural sound receptors – where they are converted into neural signals and processed by the auditory cortex.^[^ ^5^ ^]^

**Figure 1:**
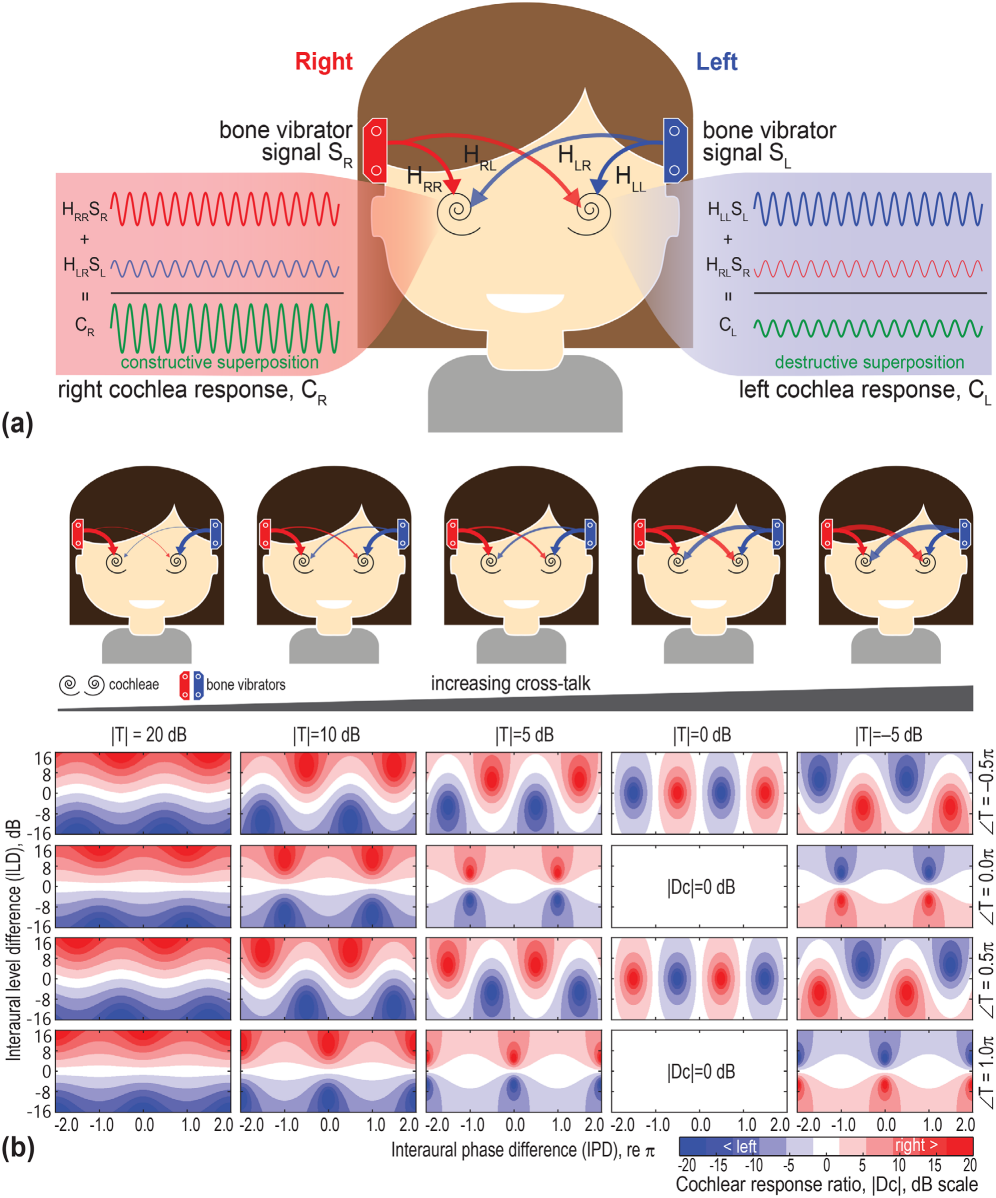
Theoretical model describing wave interference under bilateral bone conduction. a) A schematic representation of the model. For each cochlea, the response results from the summation of signals originating from both the ipsilateral and contralateral sides (red and blue colored signals). Wave interference occurs at the cochlea, leading to either constructive or destructive superposition (the green‐colored signals). b) Model‐predicted patterns of the cochlear response ratio (|*D_C_* | =  |*C_R_* |/|*C_L_* |), which depends on interaural level and phase differences of bilateral signals. |*D_C_* | is expressed on a dB scale, where values greater than 0 (shown in red on the contour map) indicate a stronger response in the right cochlea, whereas values less than 0 (shown in blue) indicate a stronger response in the left cochlea. These patterns are calculated for an ideally symmetrical skull, showing how different transcranial attenuation (|*T*|) values give rise to distinct interference patterns.

A distinctive feature of BC is that vibrations travel throughout the entire skull, allowing both the left and right cochleae to receive sound signals from bilateral sound sources.^[^ ^1^, ^3^ ^]^ This phenomenon, known as “crosstalk” (see **Figure** **1**a), is negligible in the normal air conduction (AC) pathway, where sound waves travel through the outer and middle ear before reaching the cochleae. Crosstalk is a double‐edged sword for BC. On one hand, it broadens the applications of bone conduction hearing devices (BCHDs), particularly for patients with only one functional cochlea, a condition known as single‐sided deafness (SSD).^[^ ^6^ ^]^ In such cases, the device converts sound signals from the impaired side into vibrations, which then travel through the skull to the contralateral, healthy cochlea, enabling auditory perception from both sides. On the other hand, crosstalk is considered a disruptive factor for sound localization,^[^ ^7^, ^8^ ^]^ which relies on bilateral auditory cues such as interaural level/intensity difference (ILD/IID) and interaural time/phase difference (ITD/IPD).^[^ ^9^ ^]^ These cues originate from different physical mechanisms: ILD is primarily caused by the head shadow effect, whereas ITD/IPD arise due to differences in the acoustic path lengths from the sound source to each ear.

With the growing adoption of BCHDs, BC sound localization has drawn increasing attention.^[^ ^10^, ^11^ ^]^ On one hand, bilateral BCHD fittings have been clinically shown to improve spatial hearing and speech recognition in noise.^[^ ^12^, ^13^, ^14^, ^15^ ^]^ On the other hand, several studies have reported that even with prolonged adaptation, the performance of bilateral BC users still does not reach the level of individuals with normal hearing.^[^ ^7^, ^16^ ^]^ Rigorous audiological studies have further demonstrated that, even in normal‐hearing individuals, bilateral BC stimulation differs from bilateral AC in terms of localization, speech release from masking, binaural masking level difference, and precedence effect, suggesting that the spatial cue utilization under BC is intrinsically less effective than that under AC.^[^ ^17^, ^18^ ^]^ These observations have drawn attention to crosstalk, a phenomenon unique to and nearly unavoidable in BC, and a key factor in the reduced spatial performance with bilateral stimulation.

Crosstalk originates from bilateral vibratory signal summation at the cochleae, producing interference that distorts spatial cues. This has been confirmed psychoacoustically through cancellation experiments with BC stimuli,^[^ ^19^ ^]^ and physically through intracochlear pressure measurements showing that bilateral BC signals can produce both constructive and destructive interference within the cochlea.^[^ ^20^ ^]^ Such interference evidently distorts the interaural cues (ILD and IPD) for spatial hearing. Further support comes from findings that phase‐inverted bilateral BC stimulation enhances low‐frequency cochlear responses, consistent with crosstalk‐related interference effects.^[^ ^21^ ^]^

In this study, we aim to systematically investigate how bilateral BC‐induced crosstalk alters ILD and IPD cues, by combining three levels of analysis: a theoretical model of cochlear wave interference, cadaveric measurements of skull vibrations, and psychoacoustic experiments. We analyze sound lateralization patterns under BC and AC across a wide range of ILD and IPD combinations at a finer resolution than previous studies. Our results reveal substantial differences in lateralization behavior between BC and AC conditions, which are supported by both the theoretical predictions and vibrometry data. We report atypical perceptual phenomena under bilateral BC stimulation, including non‐monotonic lateralization responses and even reversals in perceived direction. These findings collectively establish cochlear‐level wave interference as a key mechanism by which crosstalk impairs spatial hearing under bilateral bone conduction.

## 2 Results

### 2.1 Sound Lateralization Patterns Under ILD and IPD Combinations

When delivering stereo (bilateral) audio signals via headphones or earphones, even with physically impossible ILD and IPD combinations, listeners can still perceive a directional bias, a phenomenon known as lateralization.^[^ ^22^ ^]^ The overall lateralization perceptions across all ILD and IPD combinations create a contour map, which we refer to as the “pattern” in this study.

#### 2.1.1 Model Predicted Patterns

Our theoretical model (see the Experimental section) calculates the cochlear response ratio |*D_C_* | (right versus left, in dB scale) under stationary, steady‐state bilateral BC stimuli to predict the pattern. This ratio is decided by four transfer functions (*H_LL_*, *H_LR_*, *H_RL_* and *H_RR_*, see Equation 5). Figure 1b illustrates the model predicted patterns for an ideally symmetrical skull, showing variations with increasing crosstalk levels, quantified by the transcranial attenuation |*T*|.

In cases of minimal crosstalk magnitude (|*T*|  ≥  20 dB in Figure 1b), |*D_C_* | =  |*C_R_*/*C_L_* | closely approximates the ILD (i.e., |*S_R_*/*S_L_* | in Figure 1a), indicating that the cochlear magnitude ratio remains nearly identical to the stimulus magnitude ratio. As crosstalk magnitude increases (i.e., as |*T*| decreases), the pattern begins to show rippling effects (|*T*|  =  10 dB and |*T*|  =  5 dB cases). When |T| reaches 0 dB, the direction of lateralization is primarily determined by IPD, while ILD modulates the perceived strength of lateralization. Notably, the lateralization becomes most pronounced when ILD equals 0. For |*T*|  <  0 dB, a significant portion of the stimulus signal transfers to the contralateral cochlea, potentially causing a reversal along the ILD axis. In such cases, a listener may perceive the sound as biased toward one side (e.g., left), even when the actual stimulus is stronger on the opposite side (e.g., right).

#### 2.1.2 Cadaver Vibrational Patterns

**Figure 2:**
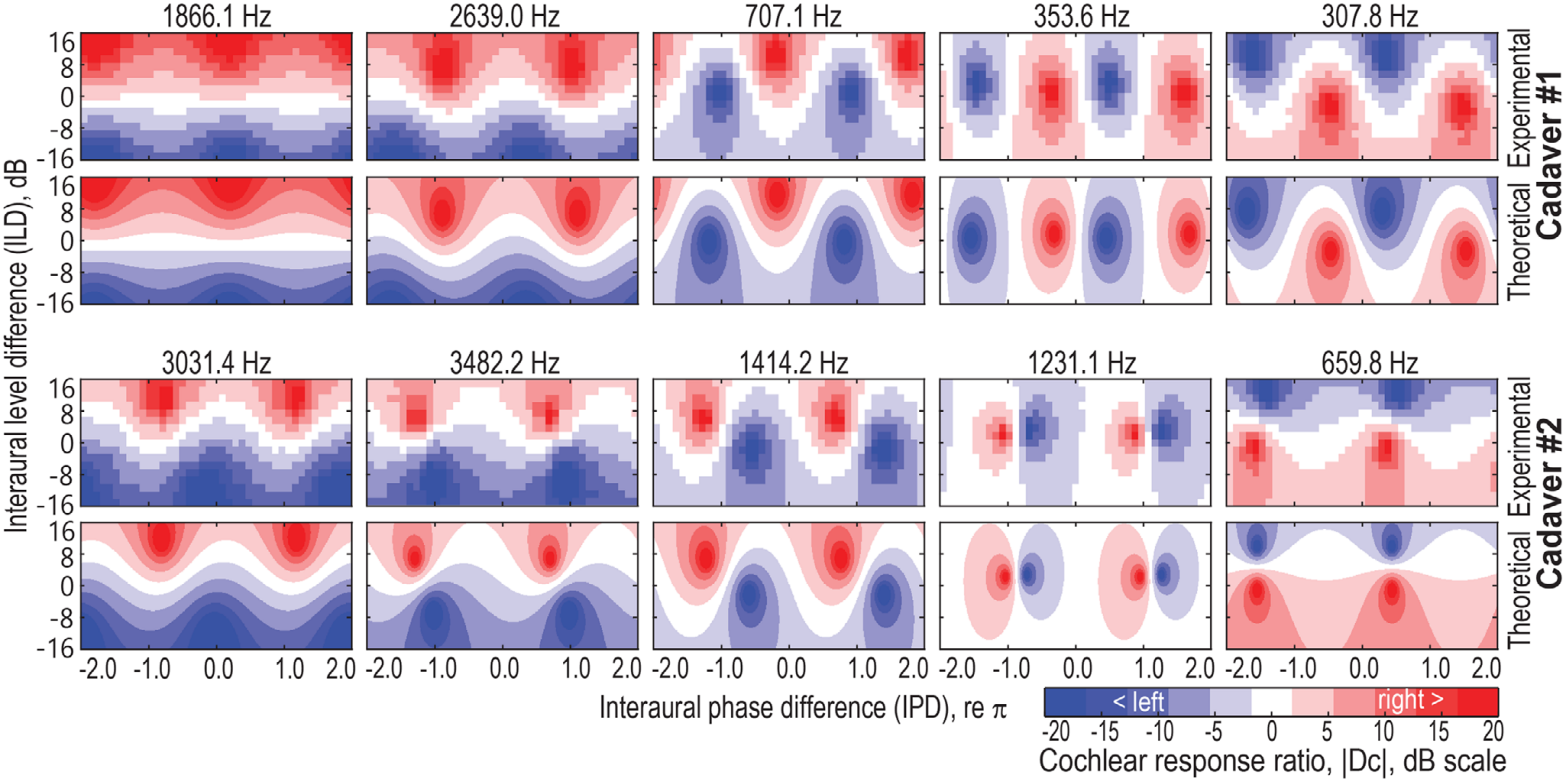
Patterns reproduced from cadaveric vibration measurements. Typical cochlear response ratio (|*D_C_* |) patterns obtained from vibration measurements in two cadaveric specimens are shown. For comparison, corresponding theoretical predictions were calculated using Equation 5, incorporating experimentally measured transfer function parameters (*H_LL_*, *H_LR_*, *H_RL_*, and *H_RR_*).

The cochlear response ratio |*D_C_* | were also estimated by measuring the vibrational velocities of both cochleae in two cadaveric specimens (see Experimental section: Vibrational measurements on cadavers), as shown in **Figure** **2**. The response ratios across different ILD and IPD combinations create distinct patterns, which vary with stimulus frequency. Additionally, theoretically predicted patterns, derived using experimentally measured parameters (*H_LL_*, *H_LR_*, *H_RL_*, and *H_RR_*, see Figure S3, Supporting Information) are presented for comparison. The predictions align well with the vibrometry results, confirming the model's accuracy. Also, the frequency‐dependent patterns closely resemble the crosstalk‐related patterns predicted by our theoretical model (refer to Figure 1).

#### 2.1.3 Psychoacoustical Experimental Revealed Patterns

**Figure 3:**
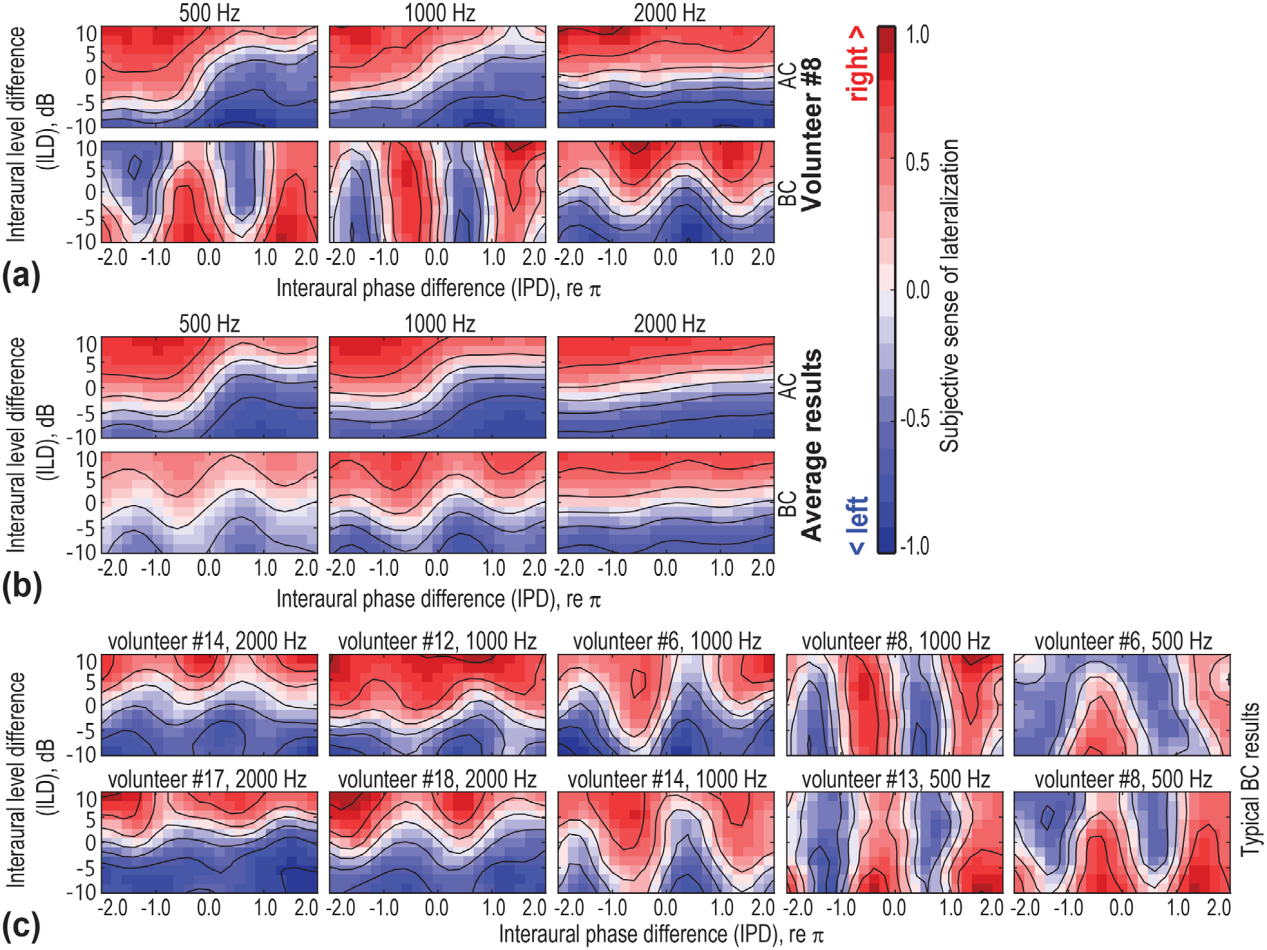
Sound lateralization patterns in psychoacoustic tests. a) Lateralization results from a representative volunteer, demonstrating a clear distinction between bone conduction (BC) and air conduction (AC) patterns. The subjective sense of lateralization is quantified on a scale from ‐1.0 to 1.0, where positive values (represented in red on the contour map) indicate a rightward bias, and negative values (represented in blue) indicate a leftward bias. b) Averaged lateralization patterns across different stimulus frequencies for both AC and BC, highlighting frequency‐dependent variations in the differences between AC and BC patterns. c) A collection of typical BC lateralization patterns from different volunteers, which align with the theoretical predictions.

The psychoacoustical lateralization judgement task (see Experimental Section: Psychoacoustical tests) can also reveal distinct patterns. **Figure** **3**a illustrates typical results from a single volunteer under bilateral AC and BC stimulations, while Figure 3b presents the average patterns from 20 volunteers. Under AC, the rules for sound lateralization are relatively simple and intuitive: the sound is always perceived as biased toward the side with higher intensity (i.e., the louder side) or the side with the leading unwrapped phase (i.e., where the phase corresponds linearly with time delay without wrapping at 2π). However, BC sound lateralization patterns exhibit greater complexity, and these simple rules do not always hold true. Additionally, as shown in Figure 3b, the differences between AC and BC lateralization patterns are more pronounced at lower frequencies (500 and 1000 Hz).

Figure 3c showcases representative BC lateralization patterns from individual volunteers. These patterns closely resemble the magnitude ratio patterns—both those theoretically predicted (see Figure 1b) and those experimentally measured (see Figure 2)—further reinforcing the model's validity.

### 2.2 Sound Lateralization with Varying IPD

Keeping ILD constant at 0 dB and gradually varying IPD (unwrapped, from −6π to +6π radians) by temporally delaying the signal to one side – i.e., introducing a corresponding ITD, which is only valid for tones with a single frequency component – the perceived sound shifts left or right accordingly. Results from our lateralization tracking task (see *Experimental Section: Psychoacoustical tests*) show that for bilateral AC tone bursts at 500 Hz, 1 kHz, and 2 kHz, all volunteers consistently reported that the sound moved smoothly from right to left. However, when the same signals were presented via BC devices, most volunteers perceived the sound as “swinging” back and forth repeatedly (refer to Table S1, Supporting Information for details).

**Figure 4:**
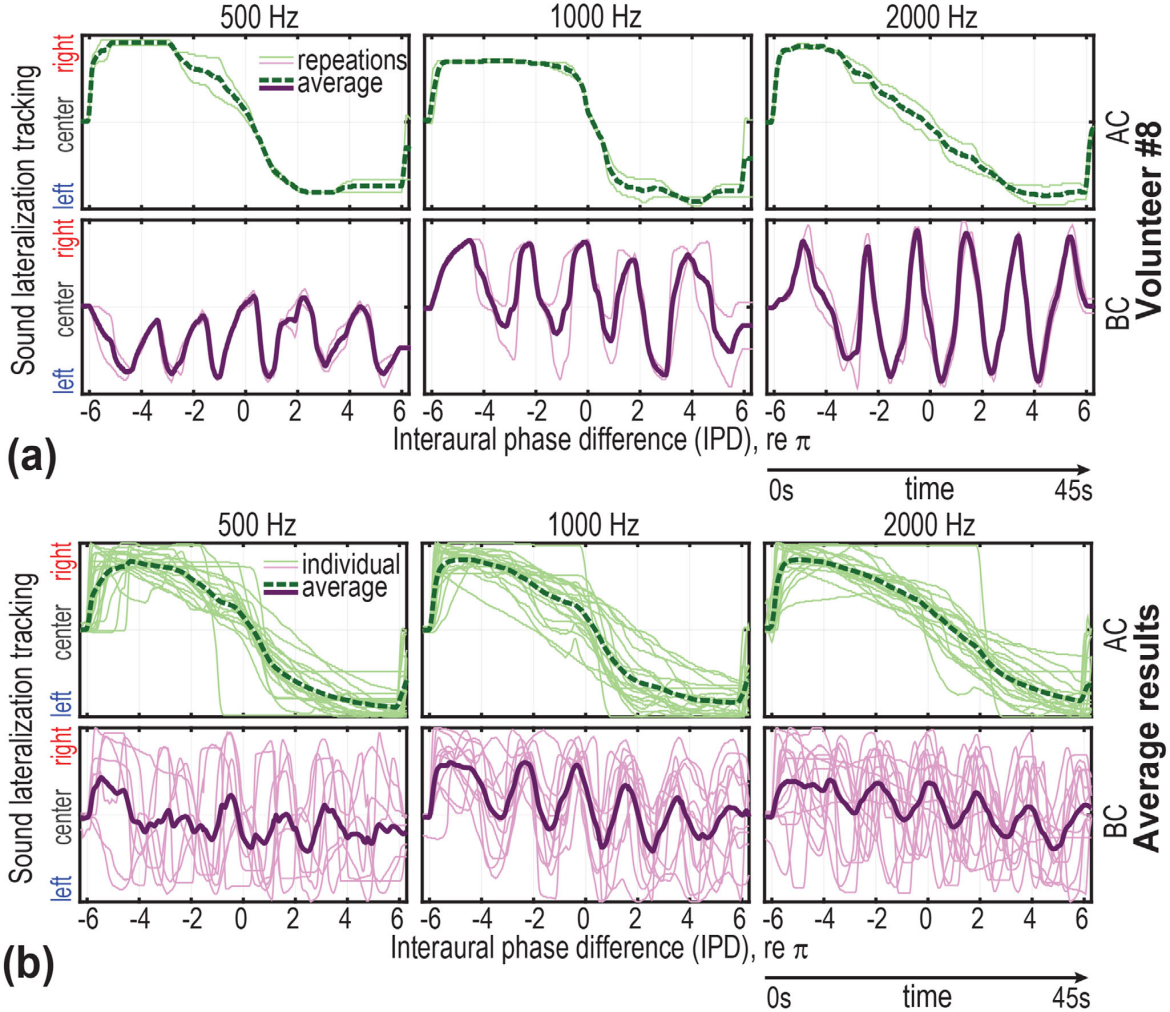
Sound lateralization tracking results. a) Tracking results from Volunteer #12. The thin curves are results for two repeated tracking task trails, and the bold curves give the average. Under air conduction (AC) stimulation (see the green dashed lines), the perceived lateralization shifts smoothly and monotonically from right to left. In contrast, under bone conduction (BC) stimulations (purple solid lines), the sound perception exhibits a back‐and‐forth “swinging” motion. b) Averaged tracking curves across all volunteers (bold lines). A similar contrast between AC and BC is observed, with AC showing a smooth lateralization shift, whereas BC demonstrates periodic fluctuations. Thin curves represent individual tracking results.

Moreover, only 50% of the volunteers successfully provided repeatable mouse‐tracking representations of their lateralization perception while simultaneously listening to the test sound. **Figure** **4**a presents typical results from Volunteer #8, while Figure 4b shows the averaged results of all volunteers who were able to provide consistent tracking. Under AC stimulation, the perceived sound moves smoothly and monotonically from right to left. In contrast, under BC stimulation, the lateralization tracking curves exhibit periodic fluctuations over time, corresponding to the varying IPD.

### 2.3 Sound Localization under AC and BC

**Figure 5:**
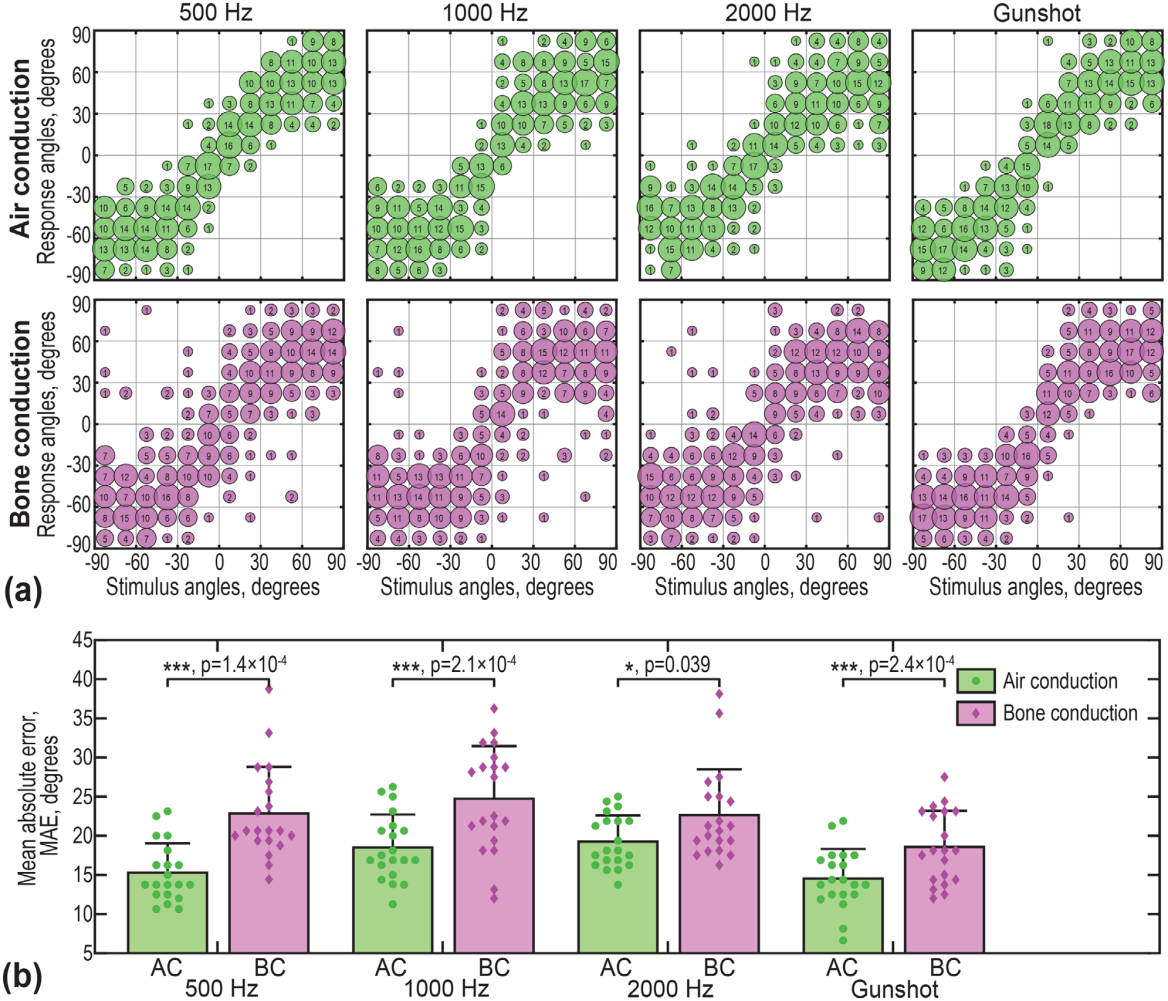
Sound localization performance. a) Stimulus‐response angle relationships, summarizing the results from all volunteers. The scatter distribution illustrates the accuracy of perceived sound direction under different conditions, with closer alignment to the y = x line indicating better localization performance. b) Mean absolute error (MAE) for the sound localization tasks, measured across different stimulus types. Data presented as mean ± SD, n = 20, P‐values are calculated using Student‐T test, NS not significant, *P < 0.05, **P < 0.01, ***P < 0.001, ****P < 0.0001. For air conduction (AC, green), the MAEs are 15.3° ± 3.7° (500 Hz), 18.5° ± 4.2° (1k Hz), 19.3° ± 3.4° (2k Hz), and 14.5° ± 3.8° (gunshot), respectively. For bone conduction (BC, purple), the MAEs are 22.8° ± 6.0° (500 Hz), 24.7° ± 6.7° (1k Hz), 22.7° ± 5.8° (2k Hz), and 18.6° ± 4.6° (gunshot), respectively.

**Figure** **5** presents the results of the sound localization task (see *Experimental Section: Psychoacoustical tests*), comparing AC and BC performances. In Figure 5a, the stimulus‐response angle relationships for all volunteers are plotted, with the *y*  =  *x* line representing perfect localization accuracy. The scatter distribution shows that AC results are generally more closely aligned with the *y*  =  *x* line, indicating more accurate sound localization. In contrast, BC data points exhibit greater deviations, suggesting reduced localization precision.

To further quantify these differences, Figure 5b displays the mean absolute errors (MAEs) across different stimulus frequencies. Repeated measures ANOVA revealed significant main effects of both stimulation type (AC versus BC) and test sound (tone bursts of 500 Hz, 1k Hz, and 2k Hz, and gunshot), with no significant interaction observed. Post hoc pairwise comparisons further confirmed that localization accuracy was consistently and significantly reduced under BC stimulation across all sound types, especially for 500, 1k Hz, and gunshot. These findings suggest that while BC allows for some degree of sound localization, it remains less accurate than AC. Furthermore, when alternative metrics such as root mean square error (RMSE) and Pearson's r were applied to the data, similar tendencies were observed (see Figure S10, Tables S4–S7, Supporting Information).

## 3 Discussion

### 3.1 BC Wave Interference and Cancellation

Although the BC peripheral auditory pathway is complex, involving multiple anatomical structures (including bones, fluids, and soft tissues), BC vibrational waves transmitted through the skull behave linearly.^[^ ^23^ ^]^ Both AC and BC sound waves reach the cochlea, generating the well‐known cochlear traveling wave^[^ ^24^, ^25^ ^]^ and eliciting auditory perception. While the mechanical processes within the cochlea differ between AC and BC (an intriguing topic in itself), the traveling waves induced by both pathways are fundamentally the same.^[^ ^26^, ^27^ ^]^ Consequently, by carefully adjusting signal level and phase, AC and BC stimuli can be made to cancel each other out, effectively minimizing the cochlear response.^[^ ^28^ ^]^ Similarly, two BC stimuli can also cancel each other through controlled superposition.^[^ ^29^ ^]^

In this study, cochlear response cancellation was achieved through the destructive superposition of signals from bilateral BC sources (see Figure 1a). When one signal is phase‐shifted by π, a constructive superposition occurs, leading to an enhanced cochlear response. According to our model calculations (Equations S4–S6, Supporting Information) and cadaveric measurements (Section S2.3, Supporting Information), cochlear response cancellation occurs at specific ILD and IPD combinations, which are determined by the frequency‐dependent BC transfer functions.

### 3.2 Sound Lateralization Patterns Under Bilateral BC

Based on a model similar to ours, Rowan and Gray^[^ ^30^ ^]^ proposed that IPD can be converted into ILD at the cochleae due to BC sound interference. We verified and further extended this theory by introducing sound lateralization patterns across varying ILD and IPD combinations under bilateral BC. In fact, the interference effect reconstructs the level and phase differences at the bilateral cochleae, making them significantly different from the original ILD and IPD cues of the bilateral BC signals. Conversely, due to the minimal crosstalk in AC, cochlear response differences under AC stimulation preserve the original ILD and IPD cues. As suggested by Stenfelt et al.,^[^ ^17^ ^]^ BC sound lateralization is likely to be predominantly influenced by the ILD at the cochleae, leading to lateralization patterns that differ significantly from those observed under AC.

This major pattern difference was confirmed in our study. These patterns, which exhibit considerable individual and frequency‐dependent variations, were consistently observed across theoretical models (see Figure 1b), cadaveric vibrometry (see Figure 2), and psychoacoustic tests (see Figure 3). Unlike AC patterns, BC sound lateralization does not always favor the side with greater stimulus level, particularly at low frequencies (see Figure 3; Figure S7, Supporting Information), nor does it change monotonically with IPD. These unconventional patterns stem from bilateral BC wave interference at the cochleae. Under bilateral stimulus with specific ILD and IPD combinations, which are inevitable in BC hearing, the response at one cochlea may be entirely canceled, leading to a perceptual bias toward the opposite side—a phenomenon predicted by our theoretical model and validated through experimental measurements (see Figure S4, Supporting Information).

### 3.3 Precedence Effect Under BC

The precedence effect, also known as the Haas effect, describes the auditory system's ability to fuse the sound image from equal‐loudness bilateral sounds (ILD = 0 dB) with an ITD, resulting in the perception of the sound being biased toward the leading side.^[^ ^31^, ^32^ ^]^ This effect has been widely applied in sound and music processing, where spatial bias can be induced simply by shifting one sound channel or delaying the other (typically within 1–40 ms) in stereo signals.

However, this technique may not function reliably under bilateral BC stimulation, particularly for steady pure‐tone signals. As revealed by our sound lateralization tracking task, sound perception under BC can “swing” periodically with varying IPD (see Figure 4). This swing effect is a direct consequence of sound wave interference, a phenomenon that has not been systematically examined before but was conceptually anticipated by Rowan and Gray.^[^ ^30^ ^]^ For complex sound signals containing multiple frequency components (such as noise, clicks, speech, or music), the constructive and destructive interference observed with pure tones is not consistent across frequencies. As a result, no clear ILD fluctuation is expected, and the swinging phenomenon typically disappears. Consequently, the precedence effect under bilateral BC is significantly weakened compared to AC.^[^ ^17^, ^18^ ^]^ This suggests that traditional stereo sound processing techniques based on AC principles may not be directly applicable to bilateral BC hearing devices.

### 3.4 Disruptions of Crosstalk on BC Sound Localization

Sound localization plays a more crucial role in daily life than one might initially assume. Beyond simply identifying the direction of a sound source, it also enhances speech recognition in noisy environments, allowing individuals to focus on a specific speaker amidst background noise.^[^ ^33^, ^34^ ^]^ While numerous clinical studies have confirmed that bilateral fitting of BCHDs improves sound localization,^[^ ^11^, ^12^, ^13^, ^14^, ^15^, ^35^ ^]^ researchers have increasingly recognized that crosstalk remains a key limiting factor, preventing further enhancement.^[^ ^7^, ^16^, ^17^, ^18^, ^36^ ^]^

MacDonald^[^ ^37^ ^]^ and Lindeman^[^ ^38^ ^]^ conducted psychoacoustic tests on normal‐hearing individuals that closely resembled our localization task. Linderman found that BC sound localization was more challenging than AC, whereas MacDonald did not. Several factors may explain this discrepancy. First, MacDonald's study had fewer participants, reducing statistical power and making it harder to detect AC‐BC differences. Second, the use of a 360‐degree sound source setup introduced reversal errors, which may have obscured differences in left‐right discrimination. Third, differences in angular resolution likely played a role. Given that AC and BC localization errors typically range between 15 and 25° (as observed in MacDonald's, Lindeman's, and our study), the coarse 45° step size used in MacDonald's and Lindeman's studies may have limited sensitivity in detecting localization differences. In contrast, the 15° step size used in our study provided higher resolution, allowing for a more precise evaluation of AC‐BC differences. The current study reveals that the MAE in BC sound localization was significantly greater than in AC (see Figure 5b). Moreover, our experimental results closely align with those of Lindeman. In Lindeman's study, the mean angular error for AC at 250, 500, and 1 kHz was 16.7°, while for BC, it was 20°.

Besides, we also observed that the AC‐BC performance gap was more pronounced at low frequencies, aligning with prior findings that crosstalk effects are more prominent in low‐frequency regions.^[^ ^36^, ^39^ ^]^ This strongly suggests that the reduced localization accuracy in BC can be attributed to crosstalk.

In theory, minimizing crosstalk could improve BC sound localization. One promising strategy involves developing signal processing algorithms designed to actively suppress crosstalk.^[^ ^40^, ^41^, ^42^ ^]^ However, crosstalk is not always detrimental. For SSD patients, a BCHD placed on the impaired side can enhance sound perception by overcoming the head shadow effect, enabling better auditory awareness.^[^ ^43^ ^]^

### 3.5 Limitations and Future Work

The mathematical model used in this study is specially designed for stable, long‐duration acoustical signals. The interference patterns of transient signals, such as impulses or rapidly changing sounds like speech, are expected to be far complex. Additionally, most of our experiments utilized tone bursts containing a single frequency component, which provides a controlled environments for studying fundamental mechanisms of BC localization and lateralization. However, how these patterns manifest under complex sounds remains an open question and will be explored in future research.

## 4 Conclusion

This study revealed wave interference patterns that demonstrate how crosstalk disrupts BC sound localization. Using theoretical modeling, cadaveric vibrometry, and psychoacoustic experiments, we found that BC lateralization patterns across varying ILD and IPD combinations differ significantly from those in AC, exhibiting inconsistencies with conventional AC lateralization rules. Additionally, BC localization accuracy is inherently lower than AC, particularly in the mid‐to‐low frequency range where crosstalk is more pronounced. These findings highlight crosstalk as a major limitation in the bilateral fitting of BCHDs, underscoring the need for crosstalk suppression strategies to improve BC spatial hearing accuracy.

## 5 Experimental Section

### The Theoretical Model

Following facts and simplifications were considered in developing the theoretical model. First, bone‐conducted vibrations travel through the skull as waves and adhere to linear dynamics.^[^ ^2^, ^3^, ^23^ ^]^ Second, the study focuses on long‐duration steady sounds, deliberately omitting rapid temporal variations. While this approach does not fully capture the complexity of real‐world auditory experiences, it provides a simplified framework for analyzing fundamental mechanisms, making the problem more tractable and enhancing theoretical clarity. Another underlying assumption of the model is that cochlear vibrations are used as an approximation of auditory perception. This approach is commonly employed in objective assessments of BC hearing.^[^ ^44^, ^45^ ^]^

The model is based on the fundamental principles of harmonic wave superposition. A harmonic vibration can be represented as a complex number *V*  = *V_r_*  + *iV_i_*, where the velocity over time is expressed as *v* (*t*) =  *V* · *e* ^2*i*π*ft*^, with *f* denoting the frequency and $i=\sqrt{-1}\;$ is the imaginary unit. The modulus $\left|V\right|=\sqrt{V_{r}^{2}+V_{i}^{2}}\;$ defines the velocity amplitude, while the phase angle is given by $\phi =\tan^{-1}\frac{V_{i}}{V_{r}}$, satisfying the relationship *V*  = |*V*|  · *e* ^*i*ϕ^. This model assumes that the vibrations of the left and right cochleae follow the equations:

(1) $$\begin{array}{c} C_{L}=S_{L}\cdot H_{LL}+S_{R}\cdot H_{RL}=\left|S_{L}H_{LL}\right|e^{i\left(\phi_{L}+\phi_{LL}\right)}+\left|S_{R}H_{RL}\right|e^{i\left(\phi_{R}+\phi_{RL}\right)} \end{array}$$

(2) $$\begin{array}{c} C_{R}=S_{L}\cdot H_{LR}+S_{R}\cdot H_{RR}=\left|S_{L}H_{LR}\right|e^{i\left(\phi_{L}+\phi_{LR}\right)}+\left|S_{R}H_{RR}\right|e^{i\left(\phi_{R}+\phi_{RR}\right)} \end{array}$$

where $S_{L}=|S_{L}|\;e^{i\phi_{L}},\;S_{R}=\left|S_{R}\right|\;e^{i\phi_{R}}$ represent the input signals from the bone vibrators placed on the left and right sides of the head, respectively. The transfer function for the signal from the left vibrator to the right cochlea is denoted as $H_{LR}=\left|H_{LR}\right|\;e^{i\phi_{LR}}$. Similarly, the transfer functions for other transmission pathways are $H_{LL}=\left|H_{LL}\right|\;e^{i\phi_{LL}}$, $H_{RL}=\left|H_{RL}\right|\;e^{i\phi_{RL}}$, and $H_{RR}=\left|H_{RR}\right|\;e^{i\phi_{RR}}$ (recall Figure 1a).

It is convenient to introduce the following notations:

(3) $$D_{S}=\left|D_{S}\right|e^{i\cdot \Delta \phi_{s}}=\frac{S_{R}}{S_{L}}$$

(4) $$D_{C}=\left|D_{C}\right|e^{i\cdot \Delta \phi_{C}}=\frac{C_{R}}{C_{L}}\;$$

where *D_S_* represents the interaural difference between the input signals, with |*D_S_* | = |*S_R_* | /|*S_L_* | corresponding to the ILD and **Δ** ϕ_*S*_ = ϕ_*R*_ − ϕ_*L*_ representing the IPD. Similarly, *D_C_* represents the difference between the responses of the bilateral cochleae, where |*D_C_* | denotes the level difference, and **Δ**ϕ_*C*_ denotes the phases difference between the cochlear responses.

Substituting Equations (1), (2), and 3 into Equation 4 yields

(5) $$D_{C}=\frac{H_{LR}+D_{S}\cdot H_{RR}}{H_{LL}+D_{S}\cdot H_{RL}}$$

and assuming perfect symmetry of the skull, where HLL=HRRΔ=HI and HLR=HRLΔ=HC, and introducing transcranial attenuation *T*  = *H_I_* /*H_C_* gives

(6) $$D_{C}=\frac{H_{C}+D_{S}\cdot H_{I}}{H_{I}+D_{S}\cdot H_{C}}=\frac{1+D_{S}\cdot T}{T+D_{S}}$$

### Vibrational Measurements on Cadavers

Two fresh‐frozen cadaver heads (both Asian males) were used for testing. The experiments were approved by the Ethics Committee of Eye & ENT Hospital of Fudan University (No.20210301). Prior to measurement, a mastoidectomy was performed on both ears to ensure direct access to the cochlear promontory, while all other structures, including the middle ear and cochleae, remained intact. Small reflective tapes (1 mm×1 mm, embedded with tiny glass balls of 50 µm diameter) were affixed to the promontory to facilitate laser Doppler vibrometry (see Figure S1, Supporting Information for the experimental setup).

The cadaver heads were steadily positioned on a metal base, ensuring stability throughout the measurement process. Two identical bone vibrators (B71, Radioear) were mounted on the skin above the auricles using a metal headband, and neither the cadavers nor the vibrators were moved during the entire experiment. Bilateral stimulus signals were generated using NI9263 (mounted on NI9178, with two channels in use, National Instruments, USA), and amplified by two identical power amplifiers (B&K 2718, Denmark; Gain: 0 dB) before being delivered to the bone vibrators. The maximum applied voltage was about 1 V.

A laser Doppler vibrometer (CLV 2500, Polytec, Germany; sensitivity: 5 mm s^−1^ V^−1^) was used to measure the velocity of both cochlear promontories. The velocity signals were acquired via an NI cDAQ system (NI9234 mounted on NI9178, National Instruments, USA; sampling rate: 51 200 Hz). Additionally, the voltage signals to the bilateral bone vibrators were also recorded. The entire measuring process was automated using an in‐house Matlab program.

The experiment was conducted in two stages. First, the transfer functions (*H_LL_*, *H_LR_*, *H_RL_*, and *H_RR_*) were determined using chirp signals (100–10 000 Hz), and tone‐bursts at 44 discrete frequencies (250–5000 Hz). Second, for each of the 44 frequency points, bilateral tone‐burst were presented with varying ILD (from ‐16 to 16 dB, in 2 dB steps) and IPD (from −2π to 2π, in 0.1π increments) combinations, the velocities of both the left and right cochlear promontories were recorded.

All the signals were analyzed using Matlab (v2023a, Mathworks, USA) with built‐in FFT processing, ensuring accurate spectral decomposition of the measured vibrations.

### Psychoacoustical Tests

Twenty young, healthy adults (15 females and 5 males) were recruited for the psychoacoustic tests, which comprised three key tasks: lateralization judgment, lateralization tracking, and sound localization. Each of the three tasks was performed under both AC and BC stimulation, with sound stimuli presented at different frequencies (500, 1k, and 2k Hz tone‐bursts). All tests were conducted in a quiet, soundproof room to minimize external auditory interference.

For AC stimulation, participants wore a pair of headphones (Sony MDR‐7506), while for BC stimulation, two bone vibrators (Radioear B71) were placed on the mastoid and secured using a metal headband. The experimental tasks were controlled via custom Matlab programs with graphical interfaces, ensuring consistency across trials. Bilateral sound signals were played through a professional external sound card (Steinberg UR44), and participants responded using a keyboard or mouse pointer.

### Lateralization Judgment Task

Participants were presented with bilateral tone bursts under controlled ILD and IPD combinations. The ILD ranged from ‐10 dB to 10 dB (2 dB intervals) and IPD ranged − 2π to +2π (0.2π interval) by shifting the onset time. The volunteers were asked to judge whether the sound was perceived as biased toward the left or right. Five choices are optional, very left, left, middle, right, and very right. By systematically repeating this task across 231 different ILD and IPD combinations with random order, we obtained lateralization contour maps, reflecting individual perceptual patterns.

### Lateralization Tracking Task

This task involved a sequence of 121 bilateral tone bursts played continuously for ≈45 s. While the ILD was kept constant at 0 dB, the IPD gradually varied throughout the trial, from an unwrapped − 6π to + 6π radians, by shifting the onset time. Participants were instructed to track their perceived lateralization in real‐time by moving a mouse cursor left or right, providing a continuous representation of their sound perception dynamics.

### Sound Localization Task

In this task, sound stimuli (tone bursts or gunshot sounds) were synthesized using the head‐related transfer function (HRTF) of a standard KEMAR mannequin to simulate sound sources positioned at different angles in the azimuthal plane in front of the participant. The participant's task was to identify the perceived direction of the sound source. By repeating this process across multiple trials, the participant's localization accuracy was quantified mainly using the mean absolute error (MAE) between the actual stimulus angle and the participant's response angle. Additionally, root mean square error (RMSE) and Pearson’ r metrics were also adopted for performance analysis.

For all tasks, sound levels were maintained at 40–50 dB above hearing threshold (dB HL) for both AC and BC stimulation. This ensured clear auditory perception while avoiding excessive loudness that could lead to auditory fatigue over extended test durations. The entire experiment lasted ≈3 to 4 h per participant, with scheduled breaks every 10 to 20 min to prevent fatigue. For a more detailed description of the psychoacoustic test setups and task protocols, refer to Sections S3.1 and S3.2 and Video S1 (Supporting Information).

### Statistical Analysis

The volunteer performance metrics in the sound localization task, such as MAE, were reported as mean ± standard deviation (SD). Repeated measures ANOVA followed by post hoc pairwise comparisons with Satterthwaite‐adjusted degrees of freedom was used to assess group differences. P‐values less than 0.05 were considered statistically significant. All statistical analyses were conducted using the Matlab (v2023a) Statistics and Machine Learning Toolbox.

## Conflict of Interest

The authors declare no conflict of interest.

## Author Contributions

L.‐J.R. and Y.Y. contributed equally to this work. Ren, Li, and Zhang designed the research project. Ren developed all the codes used in this research. Ren and Yu performed the psycho‐acoustical tests. Ren, Li, and Xie performed the cadaver vibrometry. Ren, Yu, Hua, and Liang developed the theoretical model. Ren and Yu wrote the manuscript. All authors participated in reviewing and revising the manuscript.

## Supporting information



Supporting Information

Supplemental Video 1

## Data Availability

Matlab demos of the theoretical model are available (see Section S1.1, Supporting Information), allowing readers to generate additional patterns beyond those shown in Figure 1. We also provide several stereo audio files (.wav format) with continuously varying phase differences. Interested users may experience the precedence effect using earphones or headphones, or observe the “swinging” lateralization effect using commercial bone conduction earphones (see Section S3.5, Supporting Information). The code for psychoacoustic tests and cadaveric vibrometry is in‐house. However, most individual psychoacoustic test results are included in the Supporting Information. The complete data for cadaver measurements and psychoacoustic tests are available upon request.
