# Supplementary material for: Interference Pattern Caused by Bilateral Bone Conduction Stimulation Impairs Sound Localization
Source: Adv Sci (Weinh). 2025 Jun 10;12(32):e00302. doi: 10.1002/advs.202500302 (PMC12407360; doi:10.1002/advs.202500302)
Supplement: Supplementary file 1 — Supporting Information [file ADVS-12-e00302-s002.docx]

Supporting Information

**Interference Pattern Caused by Bilateral Bone Conduction Stimulation Impairs Sound Localization**

*Liu-Jie Ren^#^, Yi Yu^#^, Cheng Hua, You-Zhou Xie, Wen-Juan Yao, Jun-Yi Liang*, Chen-Long Li*, and Tian-Yu Zhang**

L-J Ren (renliujie@fudan.edu.cn) and Y. Yu (2200009@sumhs.edu.cn) contribute equally to this work.

L-J Ren, Y-Z Xie, C-L Li, T-Y Zhang

FPRS Department / ENT Institute / NHC Key Laboratory of Hearing Medicine,

Eye & ENT Hospital of Fudan University,

Shanghai 200031, China

Email: [chenlong.li@hotmail.com](mailto:chenlong.li@hotmail.com) (Li), [ty.zhang2006@aliyun.com](mailto:ty.zhang2006@aliyun.com) (Zhang)

Y Yu

College of Medical Instruments,

Shanghai University of Medicine & Health Science,

Shanghai 201318, China

C Hua

Department of Aeronautics and Astronautics,

Fudan University,

Shanghai 200433, China

W-J Yao

School of Mechanics and Engineering Science

Shanghai University,

Shanghai 200444, China

J-Y Liang

Genomic Medicine Institute,

Cleveland Clinic Foundation,

Cleveland, OH44106, USA

Email: [Liangj3@ccf.org](mailto:Liangj3@ccf.org)

**S1. Theoretical considerations of bone conduction (BC)**

**S1.1. Demo codes**

Codes for generating additional bilateral cochlear response ratio ($\left| D_{C} \right|=|C_{R}/C_{L}|$ in dB scale) patterns are available to public on the GitHub repository: <https://github.com/willowfly/BC_pattern/> (located in ./resources/theoretical_model_demo_code/). The codes run in a MATLAB environment (MATLAB v2023a, MathWorks). The model_symmetrical.m script implements Equation 6, producing all patterns shown in Figure 1 of the paper (assuming an ideally symmetrical skull). The code includes an interactive user interface, allowing users to adjust transcranial attenuation (by modifying amplitude $|T|$ and phase $\angle T$) and observe real-time changes in $|D_{C}|$ (see Figure S1).

The model_universal.m script computes $|D_{C}|$ based on Equation 5, allowing for a more flexible representation of bilateral cochlear responses. Unlike model_symmetrical.m, which assumes an ideally symmetrical skull, this code enables users to independently adjust the amplitudes and phases of the transfer functions $H_{LL}$, $H_{RR}$, $H_{LR}$, and $H_{RL}$, providing a broader range of pattern variations.


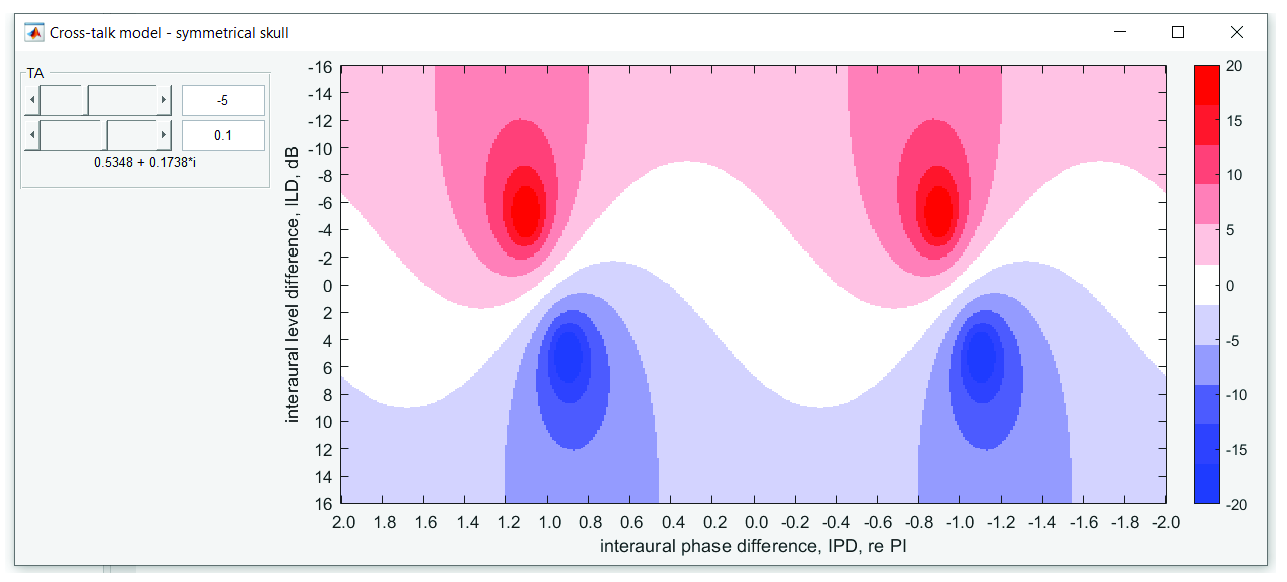


**Figure S1.** The graphical user interface of the demo code allows users to explore the impact of transcranial attenuation on $|D_{C}|$ patterns. In this example, $|T|=-5$ dB and $\angle T=0.1\pi$ were set, generating a distinct $|D_{C}|$ pattern not included in the main paper.

**S1.2. Cochlear response and its cancellation**

According to our simplified theoretical model, the vibrational response of the left and right cochleare can be expressed as:

$$\begin{aligned} \boldsymbol{C}_{\boldsymbol{L}}\mathbf{=}\boldsymbol{S}_{\boldsymbol{L}}\boldsymbol{\cdot}\boldsymbol{H}_{\boldsymbol{LL}}\mathbf{+}\boldsymbol{S}_{\boldsymbol{R}}\boldsymbol{\cdot}\boldsymbol{H}_{\boldsymbol{RL}}\mathbf{=}\left| \boldsymbol{S}_{\boldsymbol{L}}\boldsymbol{H}_{\boldsymbol{LL}} \right|\boldsymbol{e}^{\boldsymbol{i}\left( \boldsymbol{\phi}_{\boldsymbol{L}}\mathbf{+}\boldsymbol{\phi}_{\boldsymbol{LL}} \right)}\mathbf{+}\left| \boldsymbol{S}_{\boldsymbol{R}}\boldsymbol{H}_{\boldsymbol{RL}} \right|\boldsymbol{e}^{\boldsymbol{i}\left( \boldsymbol{\phi}_{\boldsymbol{R}}\mathbf{+}\boldsymbol{\phi}_{\boldsymbol{RL}} \right)}\boldsymbol{\#}\left( \mathbf{S1} \right) \end{aligned}$$

$$\begin{aligned} \boldsymbol{C}_{\boldsymbol{R}}\mathbf{=}\boldsymbol{S}_{\boldsymbol{L}}\boldsymbol{\cdot}\boldsymbol{H}_{\boldsymbol{LR}}\mathbf{+}\boldsymbol{S}_{\boldsymbol{R}}\boldsymbol{\cdot}\boldsymbol{H}_{\boldsymbol{R}\boldsymbol{R}}\mathbf{=}\left| \boldsymbol{S}_{\boldsymbol{L}}\boldsymbol{H}_{\boldsymbol{L}\boldsymbol{R}} \right|\boldsymbol{e}^{\boldsymbol{i}\left( \boldsymbol{\phi}_{\boldsymbol{L}}\mathbf{+}\boldsymbol{\phi}_{\boldsymbol{LR}} \right)}\mathbf{+}\left| \boldsymbol{S}_{\boldsymbol{R}}\boldsymbol{H}_{\boldsymbol{RR}} \right|\boldsymbol{e}^{\boldsymbol{i}\left( \boldsymbol{\phi}_{\boldsymbol{R}}\mathbf{+}\boldsymbol{\phi}_{\boldsymbol{RR}} \right)}\boldsymbol{\#}\left( \mathbf{S2} \right) \end{aligned}$$

It is possible to cancel the response of either cochlea (i.e., set $\boldsymbol{C}_{\boldsymbol{L}}\boldsymbol{=0}$ or $\boldsymbol{C}_{\boldsymbol{R}}\boldsymbol{=0}$) by adjusting the stimulus signal $\boldsymbol{S}_{\boldsymbol{L}}$ and $\boldsymbol{S}_{\boldsymbol{R}}$. To cancel $\boldsymbol{C}_{\boldsymbol{L}}$, the following conditions must be satisfied:

$$\begin{aligned} \left| S_{L}H_{LL} \right|=\left| S_{R}H_{RL} \right|\Rightarrow ILD=\frac{\left| S_{R} \right|}{\left| S_{L} \right|}=\frac{\left| H_{LL} \right|}{\left| H_{RL} \right|}\#(S3) \end{aligned}$$

$$\begin{aligned} \left( \boldsymbol{\phi}_{\boldsymbol{R}}\boldsymbol{+}\boldsymbol{\phi}_{\boldsymbol{RL}} \right)\boldsymbol{-}\left( \boldsymbol{\phi}_{\boldsymbol{L}}\boldsymbol{+}\boldsymbol{\phi}_{\boldsymbol{LL}} \right)\boldsymbol{=}\left( \boldsymbol{2}\boldsymbol{k+1} \right)\boldsymbol{\pi\Rightarrow IPD=}\boldsymbol{\phi}_{\boldsymbol{R}}\boldsymbol{-}\boldsymbol{\phi}_{\boldsymbol{L}}\boldsymbol{=}\left( \boldsymbol{2}\boldsymbol{k+1} \right)\boldsymbol{\pi+}\boldsymbol{\phi}_{\boldsymbol{LL}}\boldsymbol{-}\boldsymbol{\phi}_{\boldsymbol{RL}}\boldsymbol{\#}\left( \boldsymbol{S}\boldsymbol{4} \right) \end{aligned}$$

where $k=0,\pm1,\pm2,\ldots$ Similarly, to cancel $C_{R}$, the conditions are:

$$\begin{aligned} \left| S_{L}H_{LR} \right|=\left| S_{R}H_{RR} \right|\Rightarrow ILD=\frac{\left| S_{R} \right|}{\left| S_{L} \right|}=\frac{\left| H_{LR} \right|}{\left| H_{RR} \right|}\#(S5) \end{aligned}$$

$$\begin{aligned} \left( \phi_{R}+\phi_{RR} \right)-\left( \phi_{L}+\phi_{LR} \right)=\left( 2k+1 \right)\pi\Rightarrow IPD=\phi_{R}-\phi_{L}=\left( 2k+1 \right)\pi+\phi_{LR}-\phi_{RR}\#\left( S6 \right) \end{aligned}$$

The cancellation patterns of $C_{L}$ and $C_{R}$ determines the pattern of $|D_{C}|=|S_{R}|/|S_{L}|$. Specifically:

- $\left| D_{C} \right|\to\infty$ ($\infty$ dB) when $C_{L}$ is cancelled.
- and ${|D}_{C}|\to0$ ($-\infty$ dB) when $C_{R}$ is cancelled.

Assuming an ideally symmetrical skull, where $H_{LL}=H_{RR}\triangleq H_{I}$, $H_{LR}=H_{RL}\triangleq H_{C}$, and defining the transcranial attenuation as $T=H_{I}/H_{C}$. The cancellation conditions of $C_{L}$ becomes:

$$\begin{aligned} ILD=\frac{{|H}_{I}|}{{|H}_{C}|}=\left| T \right|, IPD=\left( 2k+1 \right)\pi+\phi_{I}-\phi_{C}=\left( 2k+1 \right)\pi+\angle T\#\left( S7 \right) \end{aligned}$$

Similarly, the cancellation conditions of $C_{R}$ are

$$\begin{aligned} ILD=\frac{\left| H_{C} \right|}{\left| H_{I} \right|}=\frac{1}{\left| T \right|}, IPD=\left( 2k+1 \right)\pi+\phi_{C}-\phi_{I}=\left( 2k+1 \right)\pi-\angle T\#\left( S8 \right) \end{aligned}$$

Therefore, whether for an ideally symmetrical skull (Equations S7 and S8) or a more general case (Equations S3-S6), it is always possible to find an appropriate (ILD, IPD) combination that cancels the response of either the left or right cochlea.

**S2. Cadaveric vibrometry: setups and results**

**S2.1. Experimental setups**

Figure S2 demonstrates the experimental setup for the cadaveric vibrometry.


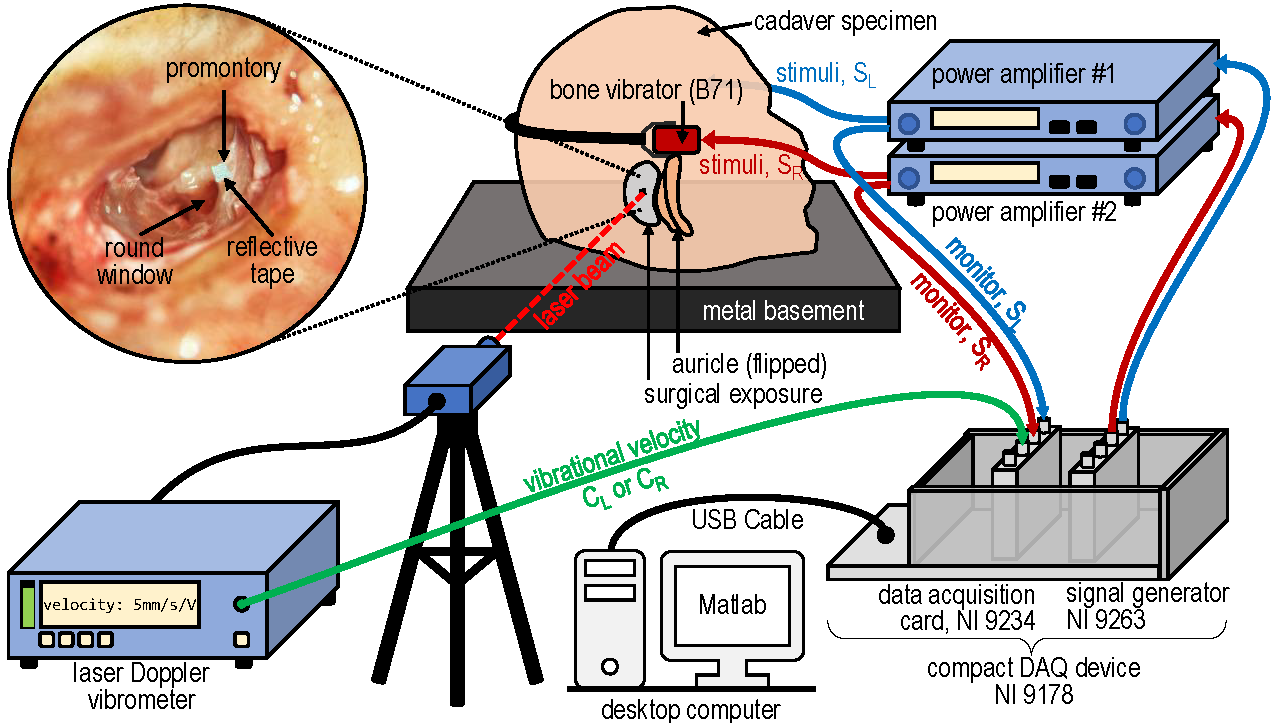


**Figure S2.** A schematic of the vibrometry setup. Two bone vibrators (B71, Radioear) are driven by two-channel signals generated by NI 9263 siginal generator, and amplified by two identical power amplifiers (Type 2718, B&K), then applied to the cadaver specimen. The vibrational velocity of the cochlear promontory is measured using a laser Doppler vibrometer (LDV, CLV-2500, Polytec). The level and phase differences of the two-channel stimulus signals are precisely controlled to investigate bilateral bone conduction interactions.

**S2.2. Results - transfer functions and transcranial attenuation**

Figure S3a presents the transfer functions of the two cadavers. The high-frequency responses of Cadaver #1 are lower than those of Cadaver #2, which can be attributed to size and weight differences (Cadaver #2 is smaller and lighter). However, the overall trends remain consistent between the two specimens. The transcranial attenuation of the cadavers can be estimated using the following equations:

$$\begin{aligned} T_{L}=\frac{H_{LL}}{H_{LR}}, T_{R}=\frac{H_{RR}}{H_{RL}}\#\left( S9 \right) \end{aligned}$$

Figure S3b shows the calculated $|T_{L}|$ and $|T_{R}|$ curves, represented by gray dashed lines. The overall transcranial attenuation $|T|$ is determined by averaging these values. For comparison, previous studies have estimated transcranial attenuation using different methodologies:

- Stenfelt (2012) estimated T through audiological measurements [1].
- Stenfelt & Goode (2005) estimated T using 3D laser Doppler vibrometry [2].

Their averaged results are also included for reference in the figure. Despite individual variability, transcranial attenuation follows a general trend. At low frequencies (below 1,000 Hz), $|T|$ is approximately 0 dB; At higher frequencies, $|T|$ increases to a range of 5-15 dB.


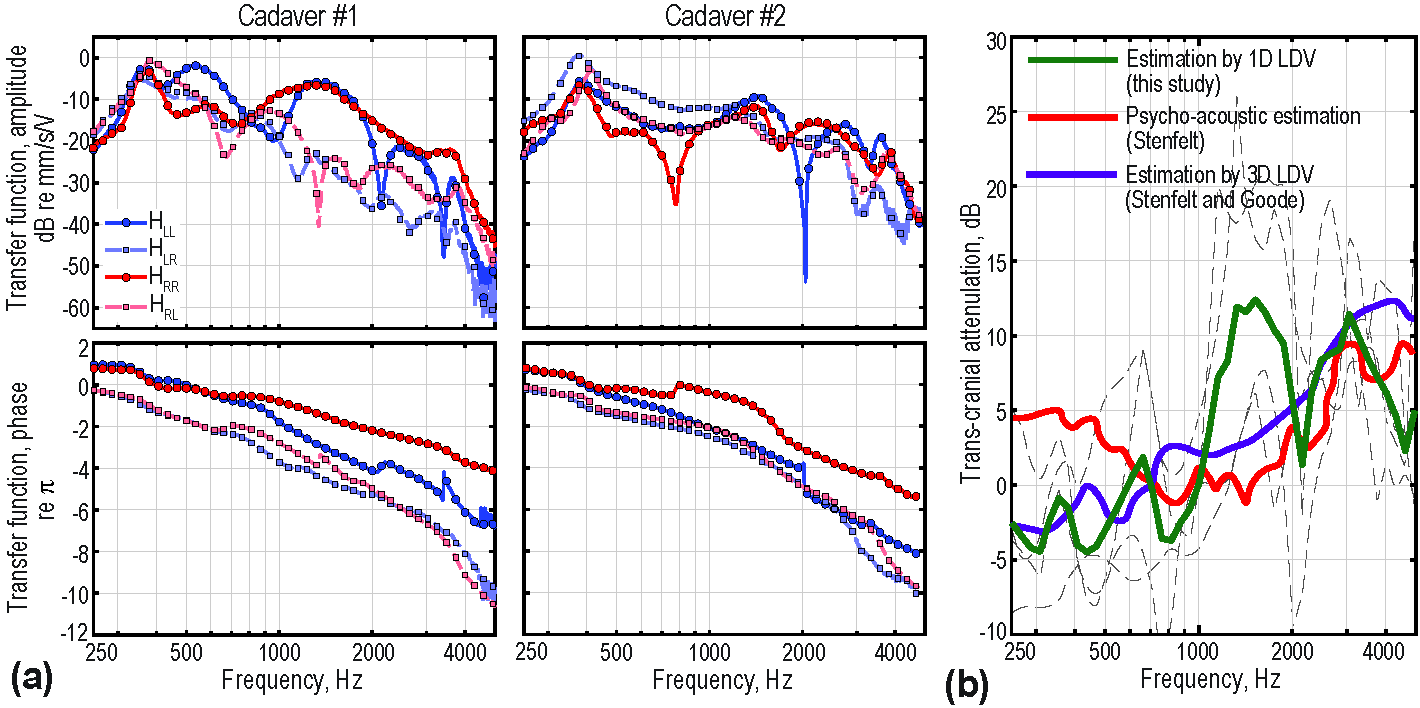


**Figure S3.** Transfer functions and transcranial attenuation calculated through cadaveric measurements. (a) Transfer functions (amplitude and phase) for the two cadavers. Each cadaver's response is represented by four curves: $H_{LL}$, $H_{RR}$, $H_{LR}$, and $H_{RL}$. (b) Transcranial attenuation estimates. The dashed lines represent calculations of $H_{LL}/H_{LR}$ and $H_{RR}/H_{RL}$ based on transfer functions, while the green bold lines show the averaged results. For comparison, the red and purple lines indicate estimations from previous literature.

1. Stenfelt, S. (2012). Transcranial attenuation of bone-conducted sound when stimulation is at the mastoid and at the bone conduction hearing aid position. Otology & neurotology, 33(2), 105-114.
2. Stenfelt, S., & Goode, R. L. (2005). Transmission properties of bone conducted sound: measurements in cadaver heads. The Journal of the Acoustical Society of America, 118(4), 2373-2391.


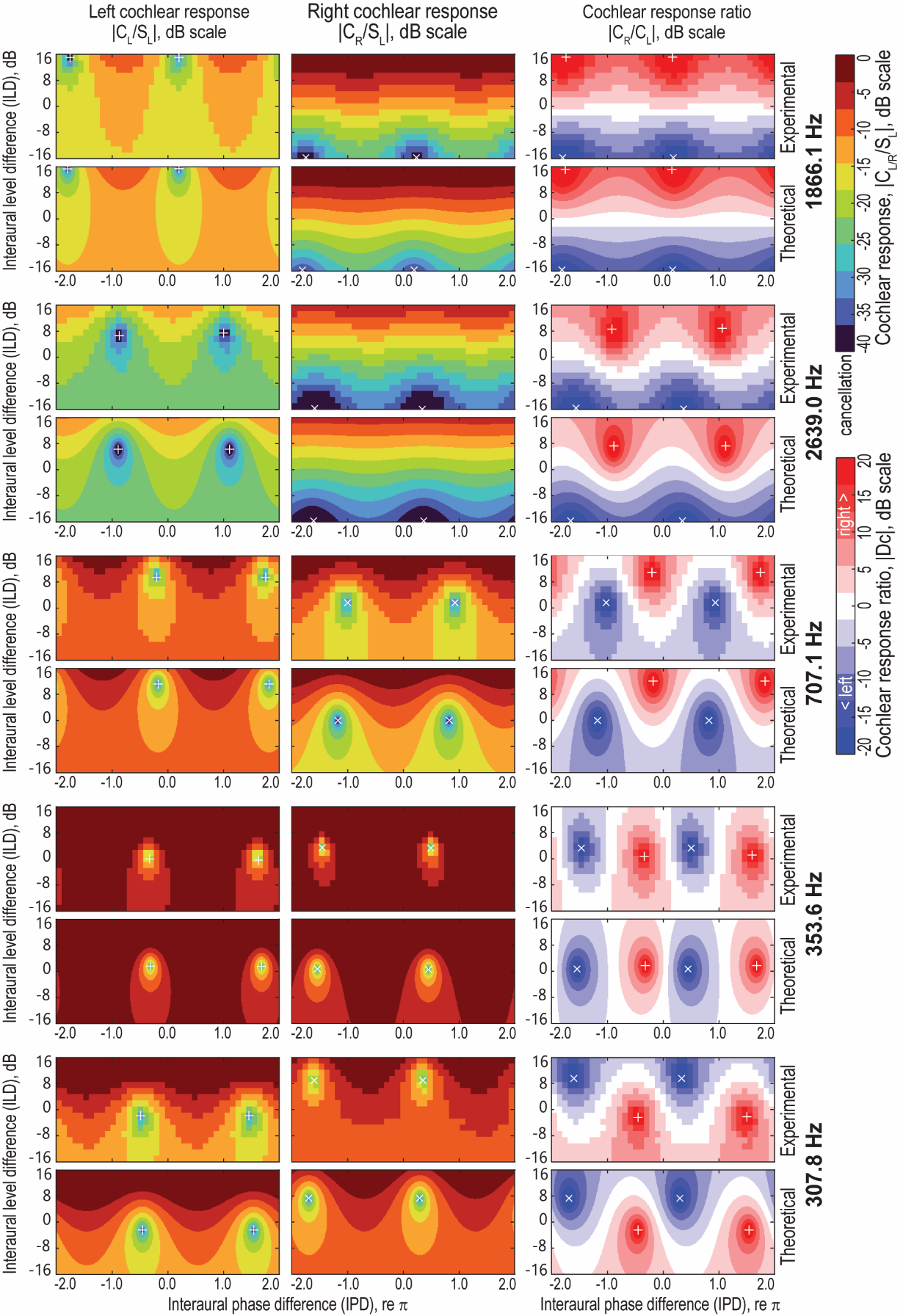


**Figure S4.** Left and right cochlear responses of Cadaver #1 across five representative frequencies. The first two columns illustrate the left ($|C_{L}|$) and right ($|C_{R}|$) cochlear responses under bilateral bone conduction (BC) stimulations at different (ILD, IPD) combinations. The third column shows the cochlear response ratio $\left| D_{C} \right|=|C_{R}/C_{L}|$. Theoretical calculations based on our model and the measured transfer functions are also plotted alongside. The "+" symbols indicate the (ILD, IPD) combinations where the $|C_{L}|$ is canceled, causing the right cochlea to dominate. Conversely, the "×" symbols mark the locations where the $\left| C_{R} \right|$ is canceled, leading to dominance of the left cochlea. These cancellation points define the poles in the $|D_{C}|$ patterns, resulting in distinct and atypical lateralization patterns.

**S2.2. Results – cochlear response and its cancellation**

Figure S4 presents the responses of the left and right cochleae ($|C_{L}|$ and $|C_{R}|$, the first two columns), along with their response ratio $|D_{C}|$ (the last column), at five representative frequencies. These results illustrate how wave interference affects cochlear responses under different interaural level differences (ILDs) and interaural phase differences (IPDs) during bilateral bone conduction (BC) stimulation.

At specific (ILD, IPD) combinations, the left and right cochlear responses are canceled, resulting in minimum sinks on the contour maps. These cancellation points (denoted as "+" for the left cochlear response, and "×" for the right cochlear response) correspond closely with the predictions from Equations S3-S6, validating our theoretical model (please refer to *Section S1.2*).

When the left cochlear response $(\left| C_{L} \right|$) is canceled, the right cochlear response ($\left| C_{R} \right|$) becomes dominant, and vice versa. This phenomenon suggests that these cancellation points serve as the poles in the cochlear response ratio ($|D_{C}|$) patterns (see the last column in Figure S4), giving rise to their distinctive and atypical characteristics.

**S3. Psycho-acoustical tests: setups and results**

**S3.1 Hardware setups for psychoacoustic tests**

Figure S5a illustrates the hardware setup used in the psychoacoustic experiments designed to assess sound lateralization and localization under bilateral BC and AC stimulations. The system is designed to ensure precise control of auditory cues, allowing for flexible manipulation of binaural signal differences (ILD and IPD).

Stereo signals with carefully controlled interaural level difference (ILD) and interaural phase difference (IPD) were generated using an external soundcard (Steinberg UR44, Yamaha Corporation) connected to the PC. For AC testing, the stereo signals were presented through a pair of high-fidelity monitor headphones (Sony MDR-7506), ensuring accurate sound transmission directly to the ears. For BC testing, the signals were delivered through two bone vibrators (B71, Radioear), which were securely positioned on the left and right mastoids using a metal headband.

**S3.2 Details of the tasks**

**S3.2.1 Lateralization judgement tasks**

This task aims to investigate sound lateralization across various (ILD, IPD) combinations under bilateral AC and BC stimulations. Tone bursts at 500 Hz, 1 kHz, and 2 kHz were used as stimuli, with ILD varying from -10 dB to 10 dB (in 2 dB intervals) and IPD ranging from -2π to 2π radians (in 0.2π intervals). For consistency, the left side was used as the reference, i.e.,

- ILD = 5 dB indicates that the right side is 5 dB louder than the left.
- IPD = π means that the right-side signal is phase-delayed by π relative to the left.

A total of 231 (ILD, IPD) combinations were randomly ordered and presented one by one. Each presentation repeated the pure-tone twice for better recognition. After each stimulus presentation, participants reported their perceived sound lateralization by selecting one of five options:

- **Very left** (-1 score)
- **Left** (-0.5 score)
- **Middle** (0 score)
- **Right** (+0.5 score)
- **Very right** (+1 score)

In this way, the test result for each volunteer was recorded as a matrix of floating-point values between -1 and 1 (denoted as $\phi_{i,j}\in[-1,1]$, where $i = 1,2,\ldots,11$ and $j = 1,2,\ldots,21$ represent ILD and IPD indices, respectively). To improve visualization and reduce noise, the matrix was smoothed twice using Laplacian smoothing.

$$\begin{aligned} \phi_{i,j}^{smooth}=\frac{1}{9}\sum_{a=-1}^{+1} \sum_{b=-1}^{+1} \phi_{i+a,j+b}\#\left( S10 \right) \end{aligned}$$

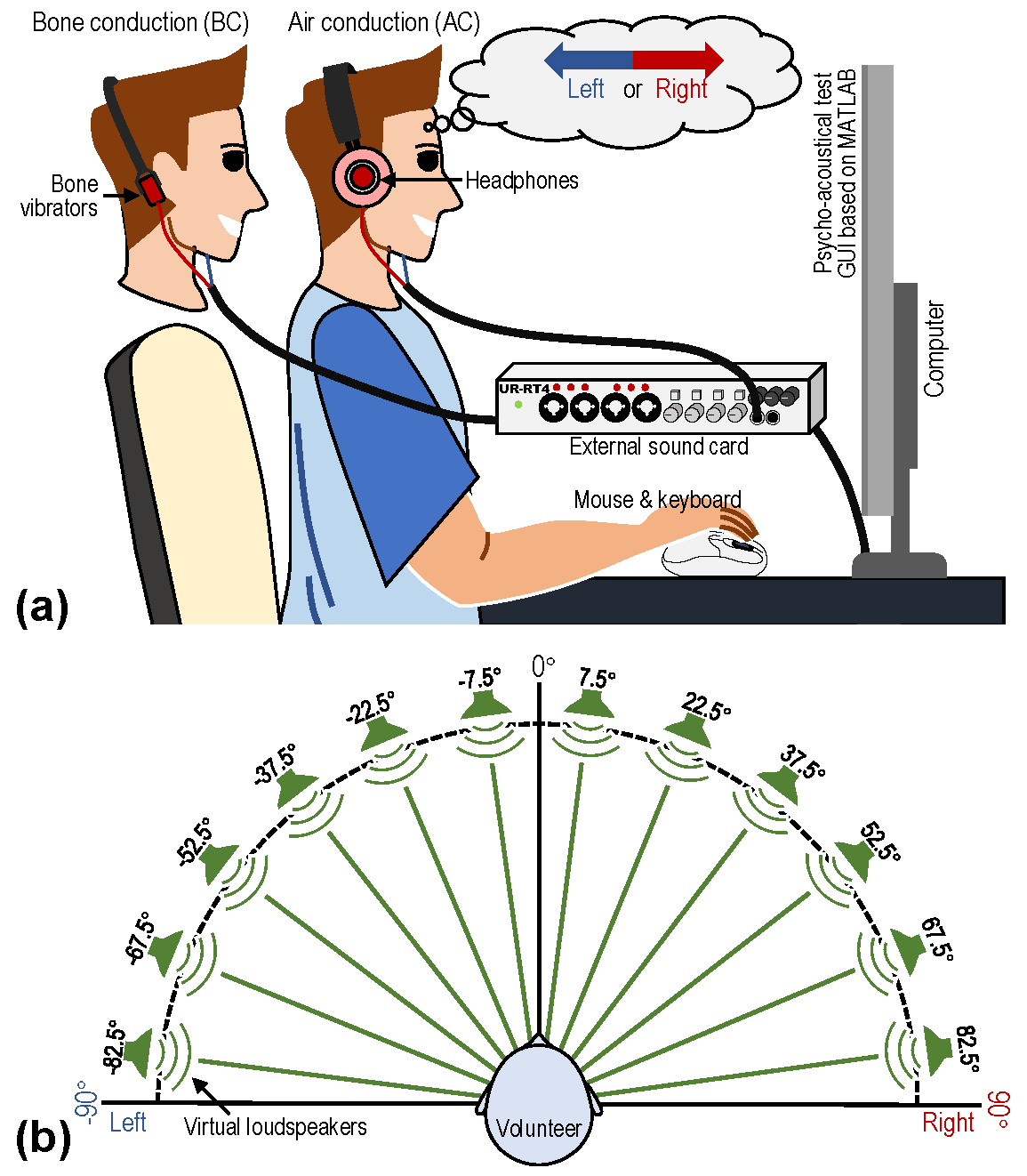


**Figure S5.** Setup of the psychoacoustic tests. (a) Hardware system. Test signals were generated by an external sound card and presented either through headphones (for air conduction) or bone vibrators (for bone conduction). Volunteers provided sound localization and lateralization responses by interacting with a graphical user interface (GUI) using a keyboard and mouse pointer. (b) Sound localization task setup. Twelve virtual sound sources were positioned in front of the volunteer. After listening to the stimulus, participants were asked to identify the perceived sound source location.

**S3.2.2 Lateralization tracking task**

The lateralization tracking task examined the effects of varying IPD on perceived lateralization under bilateral AC and BC stimulations. The stimulus consisted of a sequence of paired, two-channel tone bursts (of 500, 1k, and 2k Hz). While the ILD remained constant over time, the IPD was systematically varied from the unwrapped -6π to 6π radians in 0.1π increments, meaning the right-side signal initially led in time and gradually transitioned to a delayed position. The entire sequence contained 121 tone bursts and lasted approximately 45 seconds. In most cases, ILD was set to 0 dB; however, for certain BC conditions, ILD was adjusted within a range of -4 to +4 dB to maintain overall lateralization balance. This extended-duration stimulus was designed to investigate the precedence effect. First, volunteers listened to the signal and reported their perceived lateralization by choosing one of five options:

(A) Left to right,

(B) Right to left,

(C) Oscillating back and forth,

(D) Stationary, or

(E) None of the above.

Next, participants repeated the task while tracking their time-varying lateralization perception in real-time using a mouse pointer, moving it left and right according to their subjective perception. The pointer position was continuously recorded for analysis. Each signal type (AC or BC) and frequency (500 Hz, 1 kHz, and 2 kHz) was tested at least twice, ensuring that volunteers provided consistent, repeatable tracking results.

Since this tracking task was cognitively demanding, most volunteers required 1–3 practice trials before the final test. Despite practice, some participants were still unable to produce reliable, repeatable results, and only consistent tracking data were retained for further analysis.

**S3.2.3 Sound localization task**

This task aimed to compare sound localization performance in healthy adults under bilateral AC and BC stimulations. Four types of auditory stimuli were used: tone bursts at 500 Hz, 1 kHz, and 2 kHz, as well as a broadband gunshot sound.

The test binaural signals were synthesized based on source location, incorporating both ILD and interaural time difference (ITD, since the gunshot signal contains broadband frequency components) cues, which are essential for sound localization. The head-related transfer function (HRTF) of a standard KEMAR head was applied using the open-source Sound Field Synthesis (SFS) toolbox (version 2.5.0) to ensure accurate spatial audio reproduction.

A standard sound localization test was conducted using 12 virtual sound sources positioned in the azimuth half-plane in front of the participant (see Figure S5b). These sound sources ranged from -82.5° to 82.5° in 15° intervals. The leftmost position was set at -90°, the front at 0°, and the rightmost position at 90°. In each trial, a randomly selected synthesized sound was presented, and the participant was asked to identify the perceived direction of the sound source. Each participant completed 24 trials (N = 24), with each sound source presented twice.

The sound localization accuracy was assessed using the mean absolute error (MAE), which quantifies the discrepancy between the actual and perceived sound source locations:

$$\begin{aligned} MAE=\frac{1}{N}\sum_{i=1}^{N} \left| \theta_{i}^{\mathrm{res}}-\theta_{i}^{\mathrm{sti}} \right|\#\left( S11 \right) \end{aligned}$$

where $\theta_{i}^{sti}$ is the stimulus angle in the i-th trial, and $\theta_{i}^{res}$ is the participant’s response angle. A lower MAE indicates better localization accuracy.

Additionally, two auxiliary metrics were used to further evaluate localization performance. The root mean square error (RMSE) and Pearson’s r. RMSE is defined as

$$\begin{aligned} RMSE=\sqrt{\frac{1}{N}\sum_{i=1}^{N} \left( \theta_{i}^{\mathrm{res}}-\theta_{i}^{\mathrm{sti}} \right)^{2}}\#\left( S12 \right) \end{aligned}$$

which provides a measure of overall localization error, giving greater weight to larger deviations compared to MAE. Pearson’s Correlation Coefficient (r) is

$$r=\frac{\sum_{i=1}^{N} \left( \theta_{i}^{\mathrm{sti}}-\bar{\theta}^{\mathrm{sti}} \right)\left( \theta_{i}^{\mathrm{res}}-\bar{\theta}^{\mathrm{res}} \right)}{\sqrt{\sum_{i=1}^{N} \left( \theta_{i}^{\mathrm{sti}}-\bar{\theta}^{\mathrm{sti}} \right)^{2}}\sqrt{\sum_{i=1}^{N} \left( \theta_{i}^{\mathrm{res}}-\bar{\theta}^{\mathrm{res}} \right)^{2}}}$$

where $\bar{\theta}^{\mathrm{sti}}$ are $\bar{\theta}^{\mathrm{res}}$ are average of the stimulus and response angles, respectively. Pearson’s r evaluates the linear relationship between the actual and perceived sound source locations, $r\in[-1,1]$, with higher values indicating better consistency in localization responses.

**S3.2.4 Test procedure**

Before each task (the lateralization judgement, lateralization tracking, and sound localization task), a brief training session was conducted to familiarize participants with the experimental procedure and the computer-based user interface. Each task involved different stimulus types, including tone bursts at various frequencies and a complex pulse sound (resembling a gunshot) for the localization test. These stimuli were presented under both AC and BC conditions. To minimize order effects—such as learning effects (where performance improves as participants become more experienced) or fatigue effects (where performance declines after prolonged testing)—the presentation order of stimulus conditions and signal types was counterbalanced across participants.

The total testing duration was several hours per participant. To prevent fatigue, participants were given a mandatory rest period of at least 10–20 minutes every 10–20 minutes.

**S3.3 Test sound signals**

The stimuli used across all tests were primarily tone bursts (see Figure S6a), with a duration of approximately 200 ms (including a 10% rise and fall time) for both the lateralization judgment and localization tasks. For the lateralization tracking task, the duration of each tone burst was slightly reduced to 150 ms, though a 200 ms duration would have had minimal impact on the results. Additionally, for the localization task, a gunshot-like pulse signal was included as an alternative stimulus.

The left and right signals were presented in paired format (see Figure S6b), where ILD and IPD were precisely controlled by adjusting the amplitudes and phases of the bilateral signals. To ensure consistent audio quality, all stimuli were generated at a sampling rate of 44,100 Hz.

Figure S6c illustrates the long-duration stimulus used in the lateralization tracking task. This signal consisted of 121 pairs of tone bursts, in which bilateral amplitudes remained mostly equal, while the phase difference was gradually varied over time from $-6\pi$ to $6\pi$, with $0.1\pi$ step size. Demo audio files corresponding to these signals are available for reference (see Section S3.5).


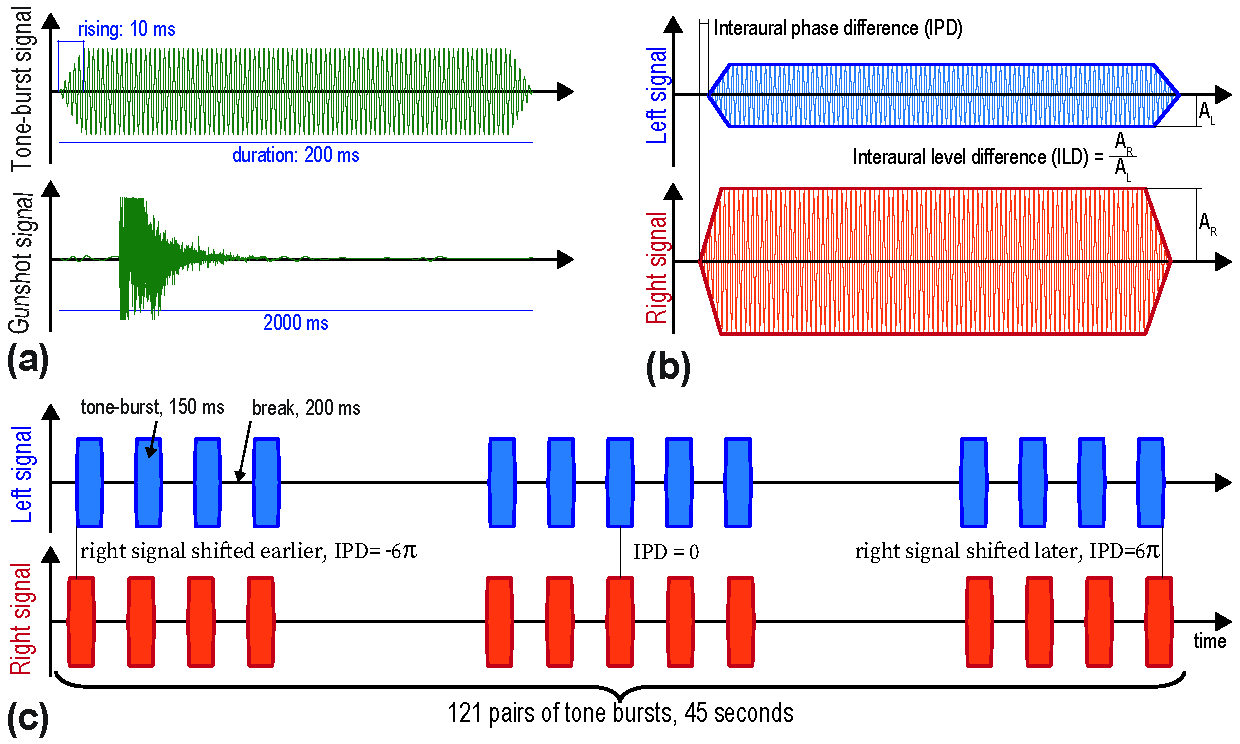


**Figure S6.** Test signals used in the psychoacoustic experiments. (a) Tone burst and gunshot signals: The primary stimuli used in the experiments, including tone bursts of different frequencies and a broadband gunshot-like pulse signal for the localization task. (b) Embedding ILD and IPD in paired tone bursts. The left and right signals were presented as paired bursts, where ILD and IPD were precisely controlled by adjusting their respective amplitudes and phases. (c) Bilateral signal for the lateralization tracking task: This stimulus consisted of 121 pairs of tone bursts, each lasting 150 ms, with a 200 ms inter-stimulus interval. The IPD between the paired signals gradually varied from -6π to 6π over time, allowing for a dynamic assessment of perceived lateralization changes.

**S3.4 Results of the psychoacoustic tests**

**S3.4.1 Basic information of the volunteers**

Table S1 presents the basic demographic information of the 20 volunteers who participated in the study. Among them, 15 were female and 5 were male. The average age of the participants was 23.2 ± 3.4 years (Volunteer #1 and #2 are the authors Ren and Yu, respectively).

**Table S1.** Basic information of the volunteers

| **ID** | 1 | 2 | 3 | 4 | 5 | 6 | 7 | 8 | 9 | 10 | 11 | 12 | 13 | 14 | 15 | 16 | 17 | 18 | 19 | 20 |
| --- | --- | --- | --- | --- | --- | --- | --- | --- | --- | --- | --- | --- | --- | --- | --- | --- | --- | --- | --- | --- |
| **Gender** | M | F | F | M | F | F | F | F | F | M | F | F | F | F | F | M | F | M | F | F |
| **Age** | 36 | 28 | 21 | 22 | 23 | 22 | 23 | 23 | 22 | 22 | 22 | 23 | 22 | 22 | 22 | 24 | 22 | 21 | 23 | 21 |

**S3.4.2 Individual results of the lateralization judgment task**

Figure S7 presents the individual results of the lateralization judgment task. Key findings from the results are summarized as follows:

1. Under AC stimulation, lateralization consistently follows the expected pattern, with sound perceived as biased toward the louder and earlier-arriving side.
2. Under BC stimulation, large individual differences were observed. In many cases, the lateralization contours differed significantly from those seen under AC stimulation.


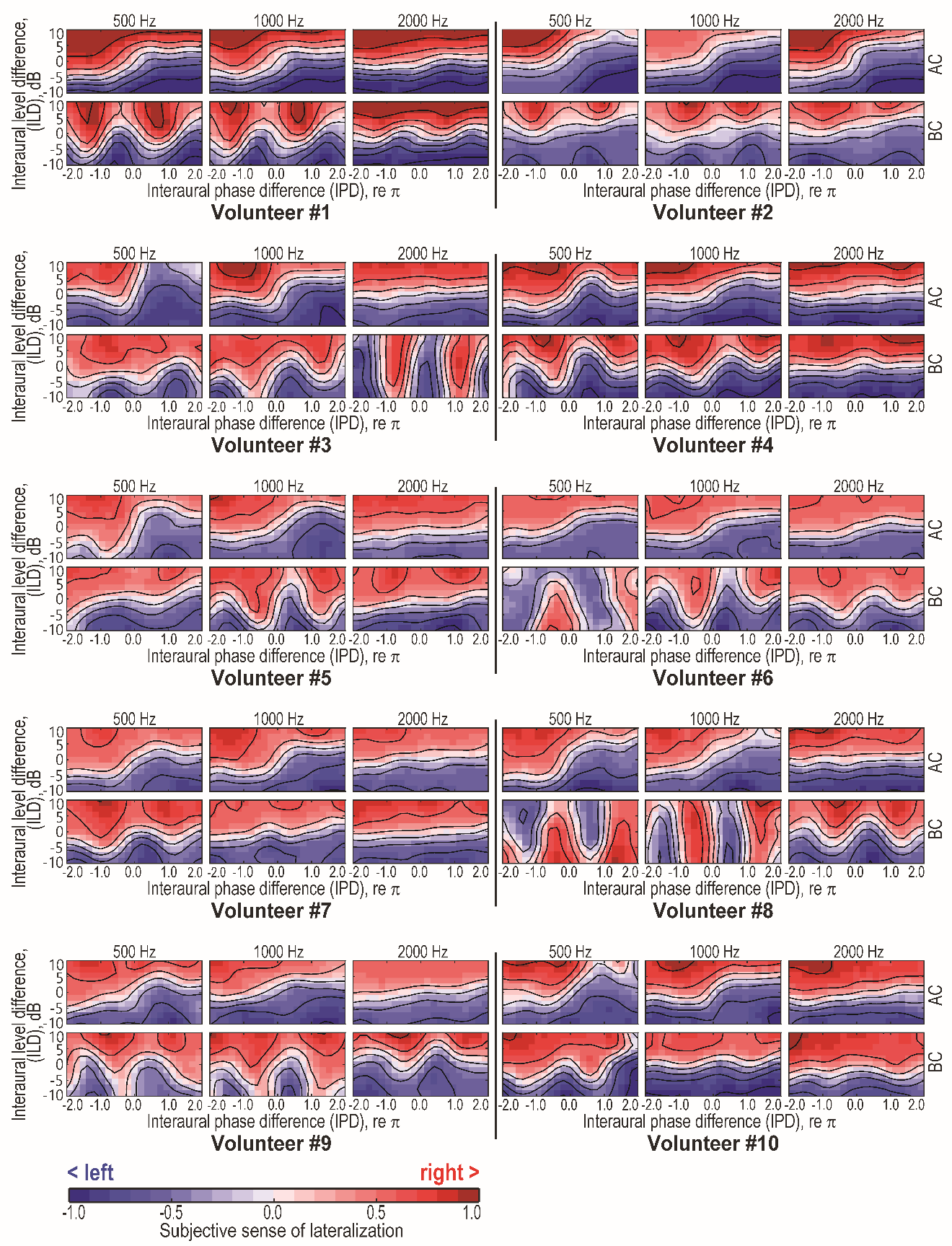


**Figure S7.** Individual results of the lateralization judgment task for 20 volunteers. Each volunteer completed the task under six different stimulus conditions, including pure tones at 500 Hz, 1 kHz, and 2 kHz, presented via AC and BC devices. The contour plots represent the volunteers’ subjective sense of lateralization, which correlates with bilateral signal differences in ILD and IPD. The results illustrate significant variability in lateralization patterns, particularly under BC stimulation, where deviations from conventional AC patterns are observed.


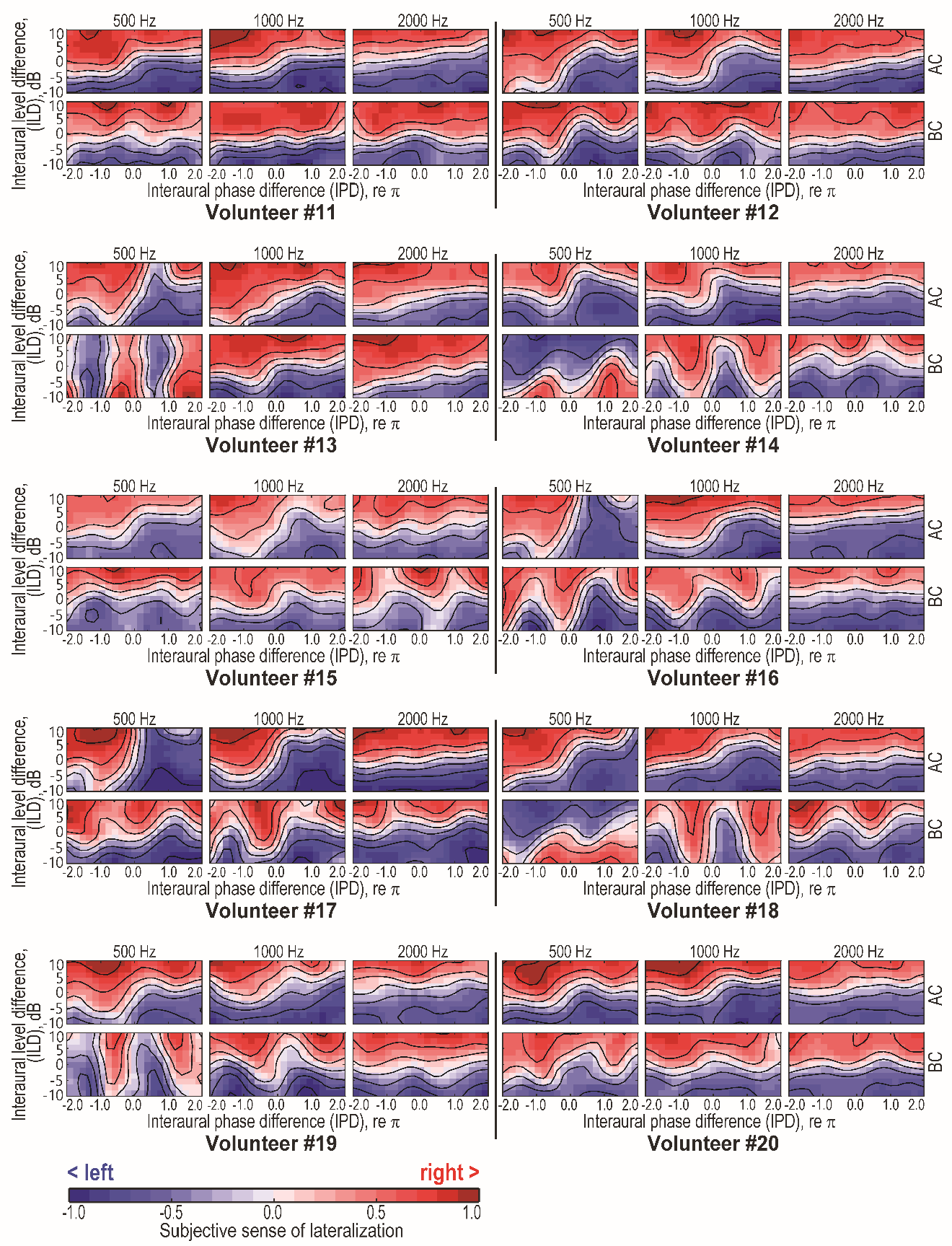


**Figure S7 (Continued).** Individual results of the lateralization judgment task for 20 volunteers.

1. Under BC stimulation, lateralization was not always biased toward the earlier-arriving side. Instead, wavy or ripple-like structures emerged in the lateralization patterns. For example, Volunteer #1 (at 500 Hz and 1 kHz) and Volunteer #3 (at 1 kHz and 2 kHz) exhibited such irregularities.
2. Under BC stimulation at low frequencies (500 Hz), some volunteers exhibited a reversal of loudness perception, where the sound was perceived as biased toward the weaker side. This phenomenon was particularly notable in Volunteers #6, #8, #14, and #18.

**S3.4.3 Results of the lateralization tracking task**

In this task, after listening to the 45-second sound, volunteers were asked to report their subjective perception of lateralization by answering the following question (originally in Chinese, translated into English):

| **Question:** *Which of the following options best describes the lateralization properties of the sound?*  A. The sound moves from left to right.  B. The sound moves from right to left.  C. The sound moves back and forth.  D. The sound does not move at all.  E. None of the above. |
| --- |

The 'ANS' columns in Table S2 present the individual responses from the volunteers. Under AC stimulation, all participants consistently reported perceiving the sound as moving from right to left (Choice B) across all three test frequencies. However, under BC stimulation, in most cases (55 out of 60 trials, or 91.7%), participants reported the sound as moving back and forth (Choice C), indicating a distinct perceptual difference between AC and BC conditions.

Additionally, the 'REP' columns indicate whether a volunteer was able to produce repeatable tracking of the sound lateralization. This task was particularly challenging under BC stimulation, as only about 50% of cases resulted in meaningful and repeatable tracking data after a brief practice session. These findings suggest that BC-induced wave interference may disrupt the stability of lateralization perception, making it difficult for participants to consistently track the perceived motion of the sound.

**Table S2.** Individual results of the volunteers to the lateralization tracking task.

| **ID** | **Air conduction** | | | | | | **Bone conduction** | | | | | |
| --- | --- | --- | --- | --- | --- | --- | --- | --- | --- | --- | --- | --- |
|  | **500 Hz** | | **1000 Hz** | | **2000 Hz** | | **500 Hz** | | **1000 Hz** | | **2000 Hz** | |
|  | ANS* | REP** | ANS | REP | ANS | REP | ANS | REP | ANS | REP | ANS | REP |
| 1 | B | Y | B | Y | B | Y | C | Y | C | Y | C | Y |
| 2 | B | Y | B | Y | B | Y | C | N | C | N | C | Y |
| 3 | B | Y | B | Y | B | Y | C | Y | C | N | C | Y |
| 4 | B | Y | B | Y | B | Y | C | N | B | N | C | N |
| 5 | B | Y | B | Y | B | Y | C | Y | C | Y | C | Y |
| 6 | B | Y | B | Y | B | Y | C | N | C | Y | C | Y |
| 7 | B | Y | B | Y | B | Y | C | N | C | Y | D | Y |
| 8 | B | Y | B | Y | B | Y | C | Y | C | Y | C | Y |
| 9 | B | Y | B | Y | B | Y | C | N | C | N | C | N |
| 10 | B | Y | B | Y | B | Y | C | N | C | N | C | N |
| 11 | B | Y | B | Y | B | Y | E | N | C | N | C | N |
| 12 | B | Y | B | Y | B | Y | C | Y | C | Y | B | Y |
| 13 | B | Y | B | Y | B | Y | C | Y | C | Y | B | Y |
| 14 | B | Y | B | Y | B | Y | C | N | C | N | C | Y |
| 15 | B | Y | B | Y | B | Y | C | N | C | N | C | Y |
| 16 | B | Y | B | Y | B | Y | C | N | C | Y | C | N |
| 17 | B | Y | B | Y | B | Y | C | N | C | Y | C | N |
| 18 | B | Y | B | Y | B | Y | C | Y | C | Y | C | Y |
| 19 | B | Y | B | Y | B | Y | C | N | C | Y | C | N |
| 20 | B | Y | B | Y | B | Y | C | N | C | N | C | Y |
| **Counts** | **A: 0**  **B: 20**  **C: 0**  **D: 0**  **E: 0** | **Y: 20**  **N: 0** | **A: 0**  **B: 20**  **C: 0**  **D: 0**  **E: 0** | **Y: 20**  **N: 0** | **A: 0**  **B: 20**  **C: 0**  **D: 0**  **E: 0** | **Y: 20**  **N: 0** | **A: 0**  **B: 0**  **C: 19**  **D: 0**  **E: 1** | **Y: 7**  **N: 13** | **A: 0**  **B: 1**  **C: 19**  **D: 0**  **E: 0** | **Y: 11**  **N: 9** | **A: 0**  **B: 2**  **C: 17**  **D: 1**  **E: 0** | **Y: 13**  **N: 7** |
| *ANS: Answers to the question for the lateralization tracking task, from A to E.  **REP: Whether the volunteer gave repeatable tracking results using a mouse pointer, Yes (Y) or No (N). | | | | | | | | | | | | |

Figure S8 presents the individual results from the sound lateralization tracking task. The data clearly demonstrate that under AC stimulation, the perceived sound consistently moves from right to left across all test frequencies and for all volunteers, providing a strong illustration of the precedence effect. However, under BC stimulation, the tracking curves exhibit substantial deviations from the AC patterns. Many volunteers reported wavy or swinging trajectories, indicating irregular and non-monotonic lateralization shifts. These observations align well with the participants' subjective responses in the lateralization perception task (see Table S2).

Additionally, not all volunteers were able to produce repeatable tracking results under BC stimulation, highlighting the instability of lateralization perception. These non-repeatable results are represented by gray lines in Figure S8. Despite their variability, these curves still show significant differences from AC tracking patterns.


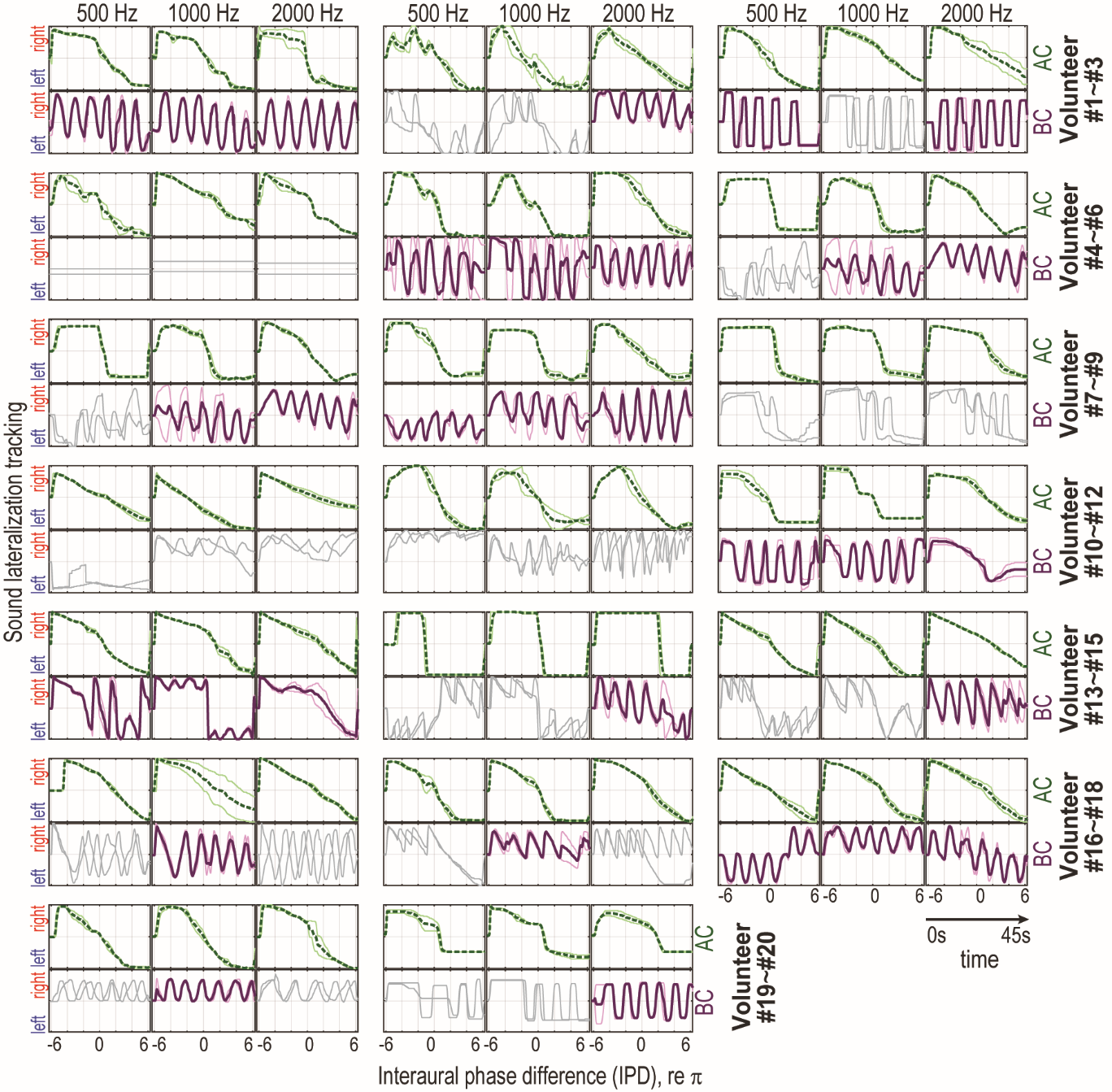


**Figure S8.** Individual results of the sound lateralization tracking task. Tracking results for 20 volunteers under six stimulus conditions (three pure-tone frequencies via AC or BC) Thin lines represent individual trials, while bold lines indicate the averaged tracking curves. Gray lines mark cases where volunteers failed to produce repeatable results, mainly under BC stimulation. Unlike the consistent right-to-left movement in AC, BC tracking curves often exhibit wavy or swinging trajectories.

**S3.4.4 Results of the sound localization task**

Figure S9 presents the individual stimulus-response results from the sound localization task, with corresponding MAE values summarized in Table S3. Under AC stimulation, the average MAE across all volunteers was 15.3° ± 3.7° (500 Hz), 18.5° ± 4.2° (1 kHz), 19.3° ± 3.4° (2 kHz), and 14.5° ± 3.8° (gunshot). Under BC stimulation, the MAE increased to 22.8° ± 6.0° (500 Hz), 24.7° ± 6.7° (1 kHz), 22.7° ± 5.8° (2 kHz), and 18.6° ± 4.6° (gunshot). Across all test signals, AC localization consistently outperformed BC localization.

Monte Carlo simulations were conducted to assess expected localization performance under different conditions. If volunteers responded completely at random, the expected MAE would be 59.6°. If volunteers could only distinguish left from right (i.e., purely lateralization without precise localization), the expected MAE would be 29.2°. The results indicate that volunteers could localize sound sources under both AC and BC conditions, though BC performance was significantly less accurate.

**Table S3.** Volunteer individual performance (MAE) of the sound localization task.

| **ID** | **Air conduction** | | | | **Bone conduction** | | | |
| --- | --- | --- | --- | --- | --- | --- | --- | --- |
|  | **500 Hz** | **1000 Hz** | **2000 Hz** | **Gunshot** | **500 Hz** | **1000 Hz** | **2000 Hz** | **Gunshot** |
| 1 | 10.625 | 11.250 | 16.250 | 8.125 | 20.625 | 13.125 | 17.500 | 12.500 |
| 2 | 12.000 | 13.808 | 15.615 | 6.615 | 14.423 | 12.000 | 18.000 | 12.000 |
| 3 | 16.250 | 16.875 | 18.125 | 14.375 | 20.625 | 36.250 | 38.125 | 23.125 |
| 4 | 11.250 | 18.125 | 23.750 | 14.375 | 23.125 | 21.250 | 17.500 | 15.000 |
| 5 | 15.000 | 15.000 | 15.625 | 17.500 | 25.625 | 19.375 | 19.375 | 16.875 |
| 6 | 10.625 | 13.750 | 13.750 | 17.500 | 20.625 | 18.125 | 21.875 | 18.125 |
| 7 | 13.750 | 16.875 | 19.375 | 13.750 | 28.750 | 18.125 | 20.000 | 17.500 |
| 8 | 20.000 | 20.000 | 21.875 | 13.750 | 18.750 | 31.875 | 21.250 | 22.500 |
| 9 | 13.750 | 17.500 | 21.875 | 12.500 | 20.625 | 33.125 | 24.375 | 18.125 |
| 10 | 13.750 | 25.000 | 21.250 | 16.875 | 26.875 | 27.500 | 16.250 | 20.000 |
| 11 | 12.500 | 14.375 | 24.375 | 13.750 | 33.125 | 30.000 | 19.375 | 13.750 |
| 12 | 16.250 | 16.250 | 16.250 | 11.250 | 19.375 | 21.250 | 18.125 | 24.375 |
| 13 | 22.500 | 25.625 | 25.000 | 21.250 | 16.250 | 21.875 | 26.875 | 23.750 |
| 14 | 20.000 | 20.625 | 23.125 | 21.875 | 38.750 | 28.750 | 19.375 | 27.500 |
| 15 | 18.125 | 16.875 | 18.125 | 12.500 | 17.500 | 21.875 | 25.000 | 23.125 |
| 16 | 12.500 | 23.125 | 17.500 | 16.250 | 28.750 | 22.500 | 20.625 | 18.125 |
| 17 | 13.750 | 20.625 | 16.875 | 16.250 | 20.000 | 28.750 | 35.625 | 13.125 |
| 18 | 23.125 | 26.250 | 17.500 | 11.875 | 23.750 | 31.875 | 21.250 | 14.375 |
| 19 | 13.750 | 21.250 | 16.875 | 12.500 | 20.000 | 28.750 | 25.000 | 14.375 |
| 20 | 16.250 | 16.875 | 21.875 | 17.500 | 19.375 | 28.125 | 27.500 | 23.125 |
| **mean** | **15.288** | **18.503** | **19.250** | **14.518** | **22.846** | **24.725** | **22.650** | **18.569** |
| **s.d.** | **3.747** | **4.208** | **3.355** | **3.788** | **5.952** | **6.723** | **5.836** | **4.619** |

RMSE and Pearson's r were also used to evaluate and compare the sound localization performance of volunteers under bilateral AC and BC stimulation. The results are presented in Table S4 (RMSE) and Table S5 (Pearson’s r). Figures S10a and S10b illustrate the statistical distributions of these two metrics. Consistently, across all three evaluation methods (MAE, RMSE, and Pearson’s r), sound localization performance under BC stimulation was significantly weaker than under AC stimulation, further confirming the impact of crosstalk-induced wave interference on BC spatial hearing.

Table S6 and S7 presents the statistical analysis results of the MAE, RMSE, and Pearson’s r metrics.


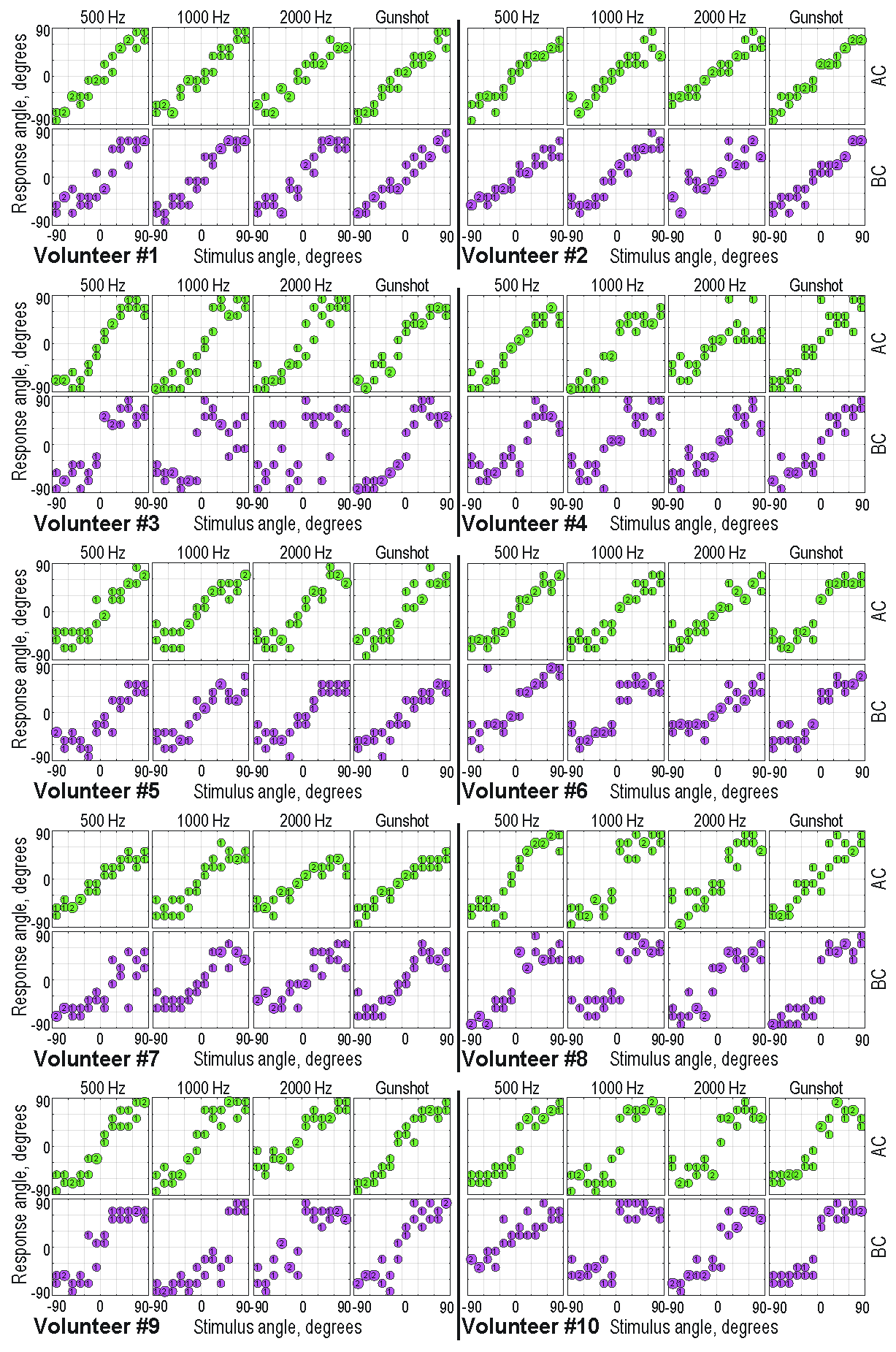


**Figure S9.** Individual results of the sound localization task. Each volunteer conducted the task under 8 different stimulus signals (pure tones of 3 frequencies and the gunshot, each was played by air or bone conduction devices). The stimulus-response angles were plotted as points.


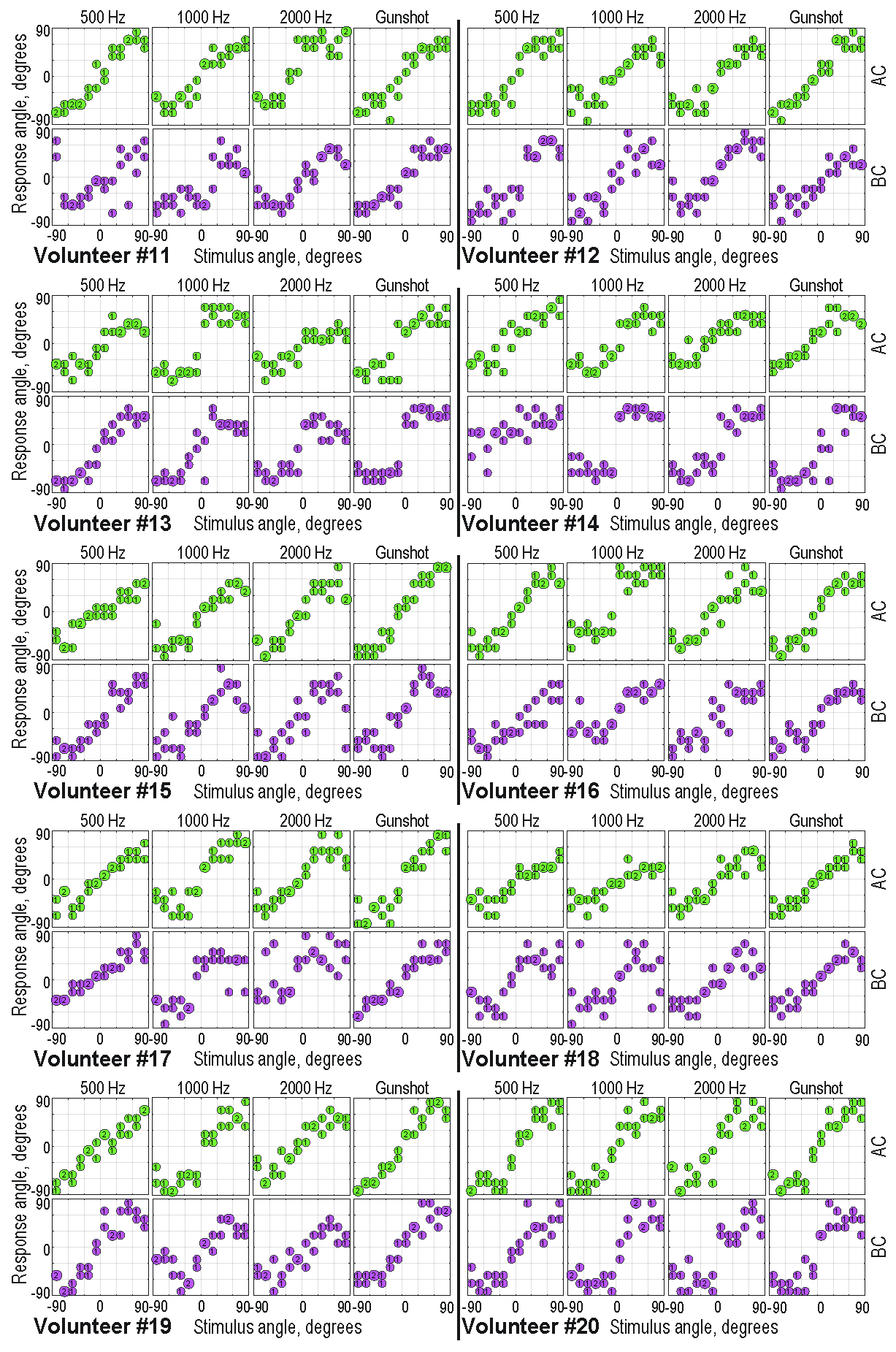


**Figure S9 (Continued).** Individual results of the sound localization task.

**Table S4.** Volunteer individual performance (RMSE) of the sound localization task.

| **ID** | **Air conduction** | | | | **Bone conduction** | | | |
| --- | --- | --- | --- | --- | --- | --- | --- | --- |
|  | **500 Hz** | **1000 Hz** | **2000 Hz** | **Gunshot** | **500 Hz** | **1000 Hz** | **2000 Hz** | **Gunshot** |
| 1 | 12.624 | 13.693 | 18.371 | 11.859 | 22.707 | 15.910 | 19.843 | 14.361 |
| 2 | 15.743 | 20.774 | 19.321 | 10.695 | 19.290 | 16.284 | 26.872 | 15.184 |
| 3 | 20.310 | 24.303 | 22.707 | 17.589 | 25.800 | 44.371 | 48.509 | 26.517 |
| 4 | 17.321 | 22.707 | 33.260 | 23.117 | 30.465 | 29.368 | 23.717 | 20.310 |
| 5 | 18.875 | 20.767 | 20.540 | 20.310 | 31.075 | 24.686 | 25.800 | 21.433 |
| 6 | 14.031 | 18.371 | 20.767 | 20.767 | 34.233 | 25.434 | 28.229 | 20.991 |
| 7 | 19.843 | 21.866 | 27.214 | 19.843 | 36.742 | 23.914 | 27.386 | 24.109 |
| 8 | 23.717 | 26.339 | 25.434 | 19.843 | 24.495 | 42.537 | 24.495 | 28.062 |
| 9 | 17.321 | 23.717 | 28.559 | 16.202 | 24.686 | 42.537 | 32.259 | 24.686 |
| 10 | 18.371 | 29.047 | 27.726 | 23.117 | 33.119 | 35.969 | 21.651 | 25.249 |
| 11 | 15.613 | 20.078 | 31.967 | 19.843 | 52.589 | 36.486 | 27.214 | 18.875 |
| 12 | 20.767 | 25.249 | 22.079 | 15.000 | 26.517 | 30.000 | 25.062 | 29.528 |
| 13 | 28.062 | 28.886 | 33.541 | 25.981 | 19.365 | 29.528 | 32.548 | 27.386 |
| 14 | 26.693 | 25.434 | 28.559 | 25.800 | 48.023 | 32.404 | 23.519 | 32.977 |
| 15 | 22.291 | 23.914 | 24.303 | 16.771 | 21.213 | 31.672 | 40.620 | 27.557 |
| 16 | 17.854 | 30.465 | 23.717 | 18.875 | 34.369 | 30.000 | 27.214 | 24.686 |
| 17 | 21.213 | 25.062 | 25.062 | 22.079 | 24.875 | 36.997 | 48.509 | 16.489 |
| 18 | 27.895 | 32.404 | 25.249 | 18.114 | 31.524 | 49.466 | 28.395 | 22.291 |
| 19 | 16.202 | 26.693 | 23.117 | 16.771 | 24.875 | 35.969 | 31.820 | 18.625 |
| 20 | 23.318 | 21.433 | 27.214 | 23.717 | 26.517 | 32.835 | 32.692 | 29.843 |
| **mean** | **19.903** | **24.060** | **25.435** | **19.315** | **29.624** | **32.318** | **29.818** | **23.458** |
| **s.d.** | **4.406** | **4.391** | **4.366** | **4.117** | **8.704** | **8.720** | **7.894** | **5.147** |

**Table S5.** Volunteer individual performance (Pearson’s r) of the sound localization task.

| **ID** | **Air conduction** | | | | **Bone conduction** | | | |
| --- | --- | --- | --- | --- | --- | --- | --- | --- |
|  | **500 Hz** | **1000 Hz** | **2000 Hz** | **Gunshot** | **500 Hz** | **1000 Hz** | **2000 Hz** | **Gunshot** |
| 1 | 0.973 | 0.966 | 0.941 | 0.974 | 0.900 | 0.954 | 0.926 | 0.964 |
| 2 | 0.956 | 0.916 | 0.957 | 0.980 | 0.957 | 0.949 | 0.857 | 0.960 |
| 3 | 0.943 | 0.934 | 0.926 | 0.945 | 0.891 | 0.654 | 0.582 | 0.913 |
| 4 | 0.947 | 0.928 | 0.770 | 0.914 | 0.818 | 0.841 | 0.889 | 0.926 |
| 5 | 0.931 | 0.920 | 0.919 | 0.922 | 0.856 | 0.885 | 0.879 | 0.931 |
| 6 | 0.965 | 0.941 | 0.929 | 0.918 | 0.824 | 0.873 | 0.879 | 0.915 |
| 7 | 0.954 | 0.912 | 0.909 | 0.961 | 0.829 | 0.893 | 0.853 | 0.891 |
| 8 | 0.922 | 0.907 | 0.882 | 0.930 | 0.906 | 0.688 | 0.890 | 0.911 |
| 9 | 0.953 | 0.933 | 0.919 | 0.961 | 0.902 | 0.844 | 0.839 | 0.913 |
| 10 | 0.942 | 0.891 | 0.862 | 0.908 | 0.871 | 0.817 | 0.920 | 0.910 |
| 11 | 0.957 | 0.924 | 0.877 | 0.925 | 0.394 | 0.776 | 0.867 | 0.935 |
| 12 | 0.923 | 0.876 | 0.910 | 0.959 | 0.888 | 0.833 | 0.890 | 0.866 |
| 13 | 0.864 | 0.849 | 0.830 | 0.869 | 0.944 | 0.845 | 0.799 | 0.874 |
| 14 | 0.902 | 0.875 | 0.905 | 0.909 | 0.662 | 0.818 | 0.894 | 0.845 |
| 15 | 0.932 | 0.911 | 0.891 | 0.970 | 0.939 | 0.815 | 0.719 | 0.857 |
| 16 | 0.942 | 0.878 | 0.889 | 0.941 | 0.861 | 0.825 | 0.876 | 0.914 |
| 17 | 0.929 | 0.886 | 0.882 | 0.928 | 0.946 | 0.717 | 0.592 | 0.956 |
| 18 | 0.894 | 0.864 | 0.892 | 0.957 | 0.794 | 0.487 | 0.844 | 0.919 |
| 19 | 0.957 | 0.902 | 0.904 | 0.961 | 0.895 | 0.730 | 0.833 | 0.936 |
| 20 | 0.934 | 0.928 | 0.869 | 0.920 | 0.885 | 0.842 | 0.829 | 0.867 |
| **mean** | **0.936** | **0.907** | **0.893** | **0.938** | **0.848** | **0.804** | **0.833** | **0.910** |
| **s.d.** | **0.026** | **0.029** | **0.041** | **0.028** | **0.126** | **0.108** | **0.095** | **0.034** |

**Table S6.** Statistical results of the sound localization performance with different evaluation metrics

| **Evaluation metrics** | **Within-subject factors** | **Sum of squres of effects** (SumSq, Error) | **Degree of freedom**  (DF, Error) | **Mean square of  effect** (MeanSq, Error) | **F value** | **P value** | **P value**  (with Greenhouse-Geisser correction) | **P value**  (with Huynh-Feldt correction) | **P value**  (with Lower-bound correction) |
| --- | --- | --- | --- | --- | --- | --- | --- | --- | --- |
| **Mean absolute error (MAE)** | Grand mean | 61112 1261.3 | 1  19 | 61112  66.385 | 920.57 | 1.47e-17 | 1.47e-17 | 1.47e-17 | 1.47e-17 |
|  | Stimulus type* | 1127  315.32 | 1  19 | 1127  16.596 | 67.908 | 1.08e-7 | 1.08e-7 | 1.08e-7 | 1.08e-7 |
|  | Test sound* | 619.66  979.95 | 3  57 | 206.55  17.192 | 12.015 | 3.36e-6 | 1.36e-5 | 3.36e-6 | 0.0026 |
|  | Interaction* | 111.21  1117.6 | 3  57 | 37.07  19.608 | 1.8906 | 0.141 | 0.157 | 0.149 | 0.185 |
| **Root mean squre error (RMSE)** | Grand mean | 1.04e5  1874.3 | 1  19 | 1.04e5  98.648 | 1054 | 4.15e-18 | 4.15e-18 | 4.15e-18 | 4.15e-18 |
|  | Stimulus type* | 1756.2  488.33 | 1  19 | 1756.2  25.702 | 68.322 | 1.03e-7 | 1.03e-7 | 1.03e-7 | 1.03e-7 |
|  | Test sound* | 1168.7  1736.4 | 3  57 | 389.58  30.464 | 12.788 | 1.69e-6 | 1.04e-5 | 2.63e-6 | 0.0020 |
|  | Interaction* | 234.39  1891.6 | 3  57 | 78.132  33.187 | 2.3543 | 0.082 | 0.106 | 0.098 | 0.141 |
| **Pearson's R** | Grand mean | 124.93  0.152 | 1  19 | 124.93  0.008 | 15655 | 3.56e-29 | 3.56e-29 | 3.56e-29 | 3.56e-29 |
|  | Stimulus type* | 0.194  0.098 | 1  19 | 0.194  0.005 | 37.705 | 6.67e-6 | 6.67e-6 | 6.67e-6 | 6.67e-6 |
|  | Test sound* | 0.116  0.266 | 3  57 | 0.039  0.005 | 8.287 | 1.15e-4 | 5.50e-4 | 2.68e-4 | 9.62e-3 |
|  | Interaction* | 0.033  0.279 | 3  57 | 0.011  0.005 | 2.248 | 0.093 | 0.117 | 0.109 | 0.150 |
| * Stimulus type: AC and BC;  Test sound: tone burst 500 Hz, tone burst 1k Hz, tone burst 2k Hz, and gunshot;  Interaction: interaction between the stimulus type and test sound | | | | | | | | | |

**Table S7.** Pair-wise P value table

| **Comparison** | **Evaluation metrics** | | | | | |
| --- | --- | --- | --- | --- | --- | --- |
|  | Mean absolute error  (MAE) | | Root mean square error (RMSE) | | Pearson’s R | |
|  | P value | Significance | P value | Significance | P value | Significance |
| AC-500 vs BC-500 | 0.00014 | *** | 0.00031 | *** | 0.00723 | ** |
| AC-1k vs BC-1k | 0.00021 | *** | 3.37e-05 | **** | 0.00020 | *** |
| AC-2k vs BC-2k | 0.03898 | * | 0.04402 | * | 0.01930 | * |
| AC-gunshot vs BC-gunshot | 0.00024 | *** | 0.00107 | ** | 0.00376 | ** |
| AC-500 vs AC-1k | 0.00398 | ** | 0.00046 | *** | 2.28e-06 | **** |
| AC-500 vs AC-2k | 0.00202 | ** | 0.00031 | *** | 0.00063 | *** |
| AC-500 vs AC-gunshot | 0.85485 | ns | 0.92091 | ns | 0.99282 | ns |
| AC-1k vs AC-2k | 0.87197 | ns | 0.62489 | ns | 0.48297 | ns |
| AC-1k vs AC-gunshot | 0.00243 | ** | 0.00019 | *** | 0.00256 | ** |
| AC-2k vs AC-gunshot | 0.00014 | *** | 6.31e-7 | **** | 3.32e-05 | **** |
| BC-500 vs BC-1k | 0.72495 | ns | 0.73088 | ns | 0.59710 | ns |
| BC-500 vs BC-2k | 0.99972 | ns | 0.99990 | ns | 0.98017 | ns |
| BC-500 vs BC-gunshot | 0.06428 | ns | 0.04113 | * | 0.17445 | ns |
| BC-1k vs BC-2k | 0.46635 | ns | 0.62044 | ns | 0.67059 | ns |
| BC-1k vs BC-gunshot | 0.00413 | ** | 0.00078 | *** | 0.00253 | ** |
| BC-2k vs BC-gunshot | 0.06521 | ns | 0.03028 | * | 0.01524 | * |


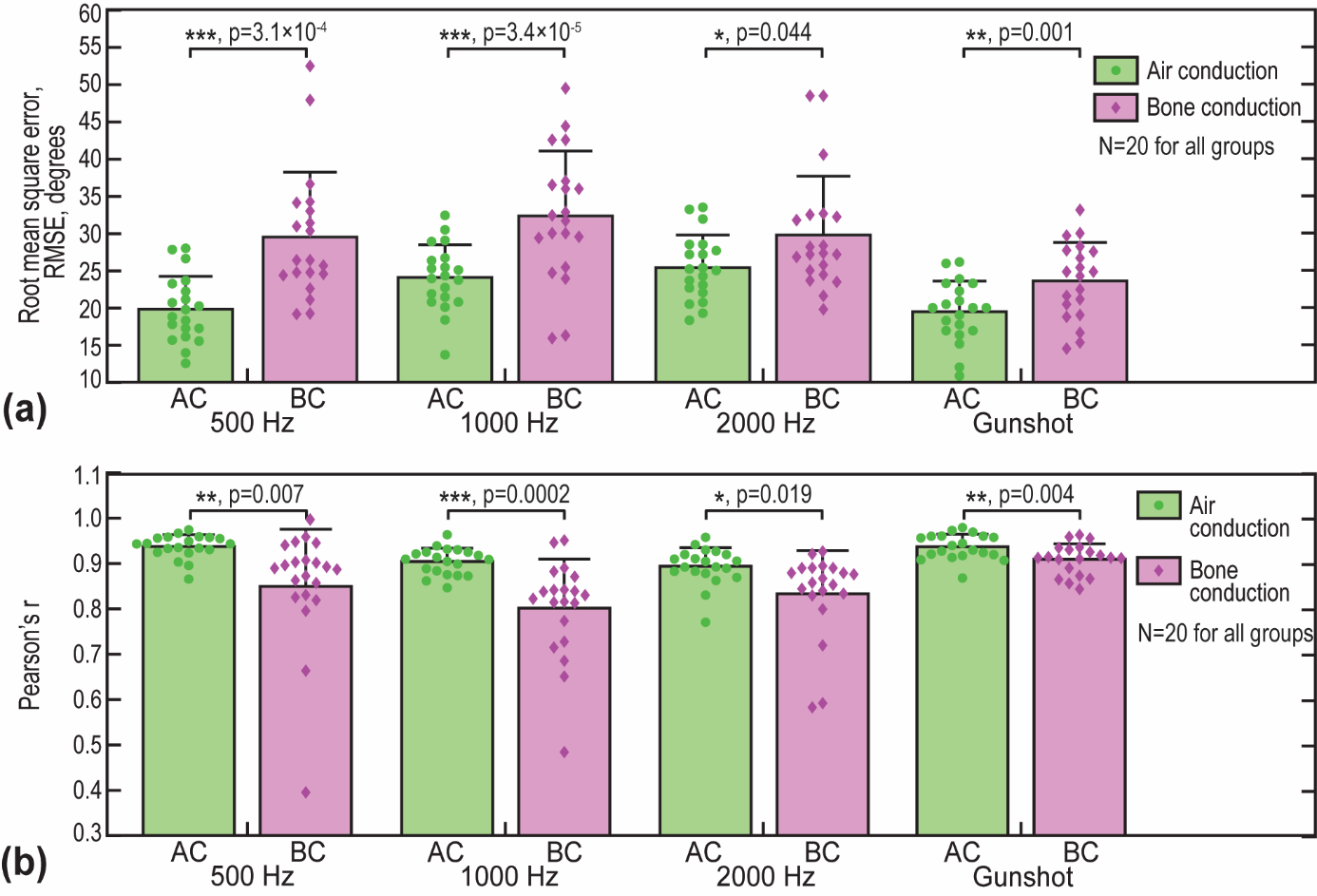


**Figure S10.** Sound localization performance of the volunteers (N=20), evaluated using RMSE and Pearson’s r. Data presented as mean ± SD, n=20, P-values are calculated using Tukey Posthoc , *P<0.05, **P<0.01, ***P<0.001. (a) RMSE results. For air conduction (AC, green), the RMSEs are 19.9° ± 4.4° (500 Hz), 24.1° ± 4.4° (1k Hz), 25.4° ± 4.4° (2k Hz), and 19.3° ± 4.1° (gunshot), respectively. For bone conduction (BC, purple), the RMSEs are 29.6° ± 8.7° (500 Hz), 32.3° ± 8.7° (1k Hz), 29.8° ± 7.9° (2k Hz), and 23.5° ± 5.1° (gunshot), respectively. (b) Pearson’s r. For AC (green), the correlation coefficients are 0.936 ± 0.026 (500 Hz), 0.907 ± 0.029 (1k Hz), 0.893 ± 0.041 (2k Hz), and 0.938 ± 0.028 (gunshot), respectively. For BC (purple), the coefficients are 0.848 ± 0.126 (500 Hz), 0.804 ± 0.108 (1k Hz), 0.833 ± 0.095 (2k Hz), and 0.910 ± 0.034 (gunshot), respectively.

**S3.5 Demos for Lateralization Tracking**

**S3.5.1 Demo audio files**

Demo audio files used in the lateralization tracking task (see S3.2.2) are available on the GitHub repository: <https://github.com/willowfly/BC_pattern/> (in ./resources/demo_audio_files/). Multiple audio files are provided, each containing a sequence of pure tones at different frequencies. In addition to the primary test frequencies 500 Hz, 1 kHz, and 2 kHz, additional files for 750 Hz, 1.5 kHz, and 3 kHz are also included.

**S3.5.2 Usage Notes**

Interested readers are encouraged to listen to the demo audio files in a quiet environment. It is recommended to use a personal desktop for playback rather than mobile devices, as the latter have not been tested for accuracy.

- For AC stimulus, any type of wired or wireless earphones/headphones is suitable.
- For BC stimulus, a pair of bone conduction earphones is required.

Figure S11a shows an example of a commercial bone conduction earphone (Bluetooth-enabled) that can be used for experiencing the demo audios. However, it is important to note that many bone conduction earphones include both a speaker and a vibrator on each side, allowing additional AC hearing, which may enhance sound perception. To minimize AC interference and achieve purer BC stimulation, we recommend:

1. Positioning the vibrators behind the auricles (on the mastoid) instead of their standard pre-auricular placement, effectively reducing AC signal effects (see Figure S11b).
2. Placing the vibrators above the auricles as an alternative positioning.
3. Applying slight pressure on the vibrators with fingers to enhance BC transmission efficiency.

Before starting the test, adjust your device volume to a comfortable listening level. Under AC stimulation, the sound should be perceived as moving from right to left (if it moves in the opposite direction, please check whether the earphones are worn correctly). Under BC stimulation, there is a high chance of perceiving a "swinging" effect, where the sound moves back and forth between left and right. Since the phase varies from -6π to 6π, the sound image will shift periodically for six complete cycles.


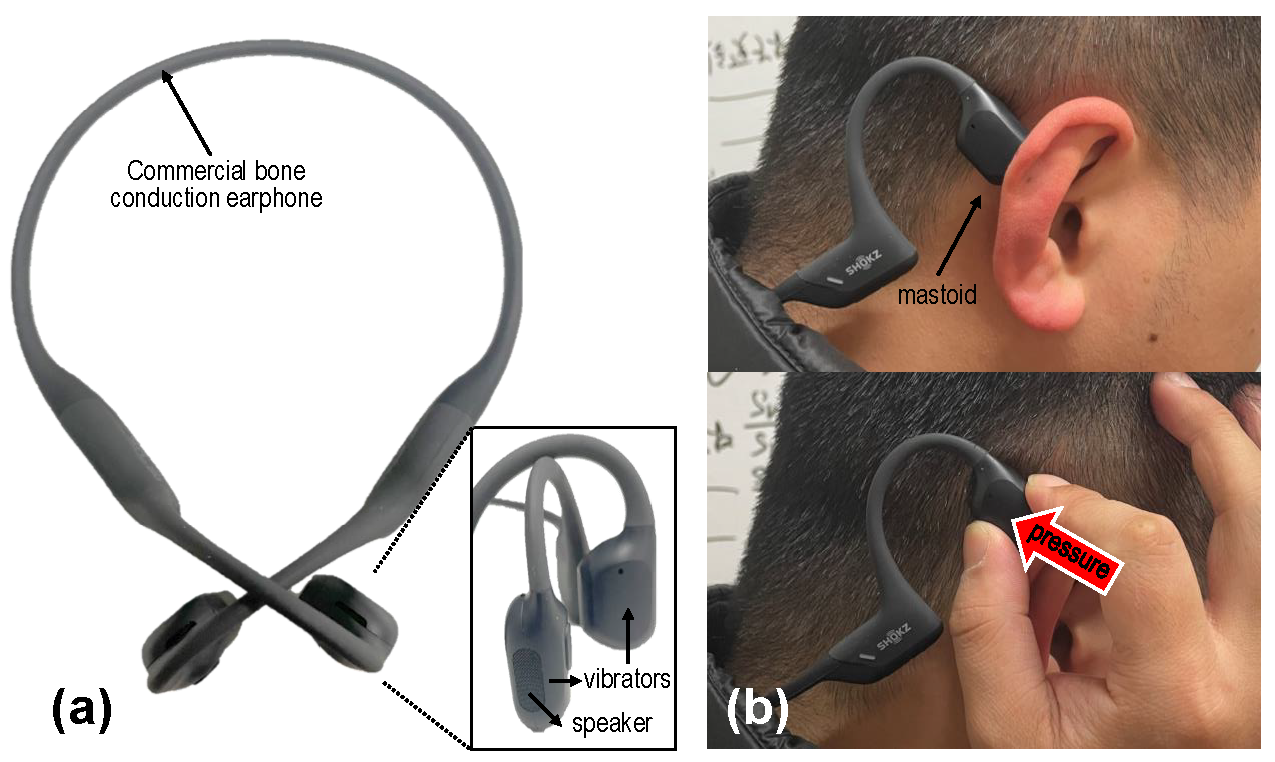


**Figure S11.** Recommended usage of bone conduction earphones for the demo. (a) A commonly available commercial bone conduction (BC) earphone, suitable for experiencing the demo audio files.

(b) Suggested wearing method to optimize BC perception while minimizing air conduction (AC) interference. Instead of placing the device at the pre-auricular area (as designed), positioning the vibrators on the mastoid behind the auricles is recommended. Additionally, applying a static pressure of 2.5–5N can enhance BC transmission, improving sound perception.
